# *Anthriscus sylvestris*—Noxious Weed or Sustainable Source of Bioactive Lignans?

**DOI:** 10.3390/plants13081087

**Published:** 2024-04-12

**Authors:** Sanja Berežni, Neda Mimica-Dukić, Gianniantonio Domina, Francesco Maria Raimondo, Dejan Orčić

**Affiliations:** 1 Department of Chemistry, Biochemistry and Environmental Protection, Faculty of Sciences, University of Novi Sad, Trg Dositeja Obradovića 3, 21000 Novi Sad, Serbia; neda.mimica-dukic@dh.uns.ac.rs (N.M.-D.); dejan.orcic@dh.uns.ac.rs (D.O.); 2 Department of Agricultural, Food and Forest Sciences, University of Palermo, Viale delle Scienze, bldg. 4, 90128 Palermo, Italy; gianniantonio.domina@unipa.it; 3 PLANTA/Center for Research, Documentation and Training, Via Serraglio Vecchio 28, 90123 Palermo, Italy; raimondo@centroplantapalermo.org

**Keywords:** wild chervil, chemical composition, anti-inflammatory activity, antiproliferative activity, structure–activity relationship

## Abstract

*Anthriscus sylvestris* (L.) Hoffm. (Apiaceae), commonly known as wild chervil, has gained scientific interest owing to its diverse phytochemical profile and potential therapeutic applications. The plant, despite being categorized as a noxious weed, is traditionally used in treating various conditions like headaches, dressing wounds, and as a tonic, antitussive, antipyretic, analgesic, and diuretic. Its pharmacological importance stems from containing diverse bioactive lignans, especially aryltetralins and dibenzylbutyrolactones. One of the main compounds of *A. sylvestris*, deoxypodophyllotoxin, among its wide-ranging effects, including antitumor, antiproliferative, antiplatelet aggregation, antiviral, anti-inflammatory, and insecticidal properties, serves as a pivotal precursor to epipodophyllotoxin, crucial in the semisynthesis of cytostatic agents like etoposide and teniposide. The main starting compound for these anticancer medicines was podophyllotoxin, intensively isolated from *Sinopodophyllum hexandrum*, now listed as an endangered species due to overexploitation. Since new species are being investigated as potential sources, *A. sylvestris* emerges as a highly promising candidate owing to its abundant lignan content. This review summarizes the current knowledge on *A. sylvestris*, investigating its biological and morphological characteristics, and pharmacological properties. Emphasizing the biological activities and structure–activity relationship, this review underscores its therapeutic potential, thus encouraging further exploration and utilization of this valuable plant resource.

## 1. Introduction

*Anthriscus sylvestris* (L.) Hoffm. (Apiaceae) is a renowned plant due to its medicinal properties. Among the closely related members of the same family there are anise (*Pimpinella anisum*), carrot (*Daucus carota*), celery (*Apium graveolens*), chervil (*Anthriscus cerefolium*), coriander (*Coriandrum sativum*), cumin (*Cuminum cyminum*), fennel (*Foeniculum vulgare*), hemlock (*Conium maculatum*), and parsley (*Petroselinum crispum*) [1]. *A. sylvestris* is the most common species of the genus *Anthriscus*.

It naturally occurs in the temperate regions of Eurasia. Although it is common in most European countries, its occurrence in the Mediterranean region is limited [2]. It is naturalized in Iceland, the Faroe Islands and Greenland [3], in North America, Canada, central and southern Africa, and in New Zealand [4,5,6]. Various authors have provided differing enumerations of accepted species within this genus. Hiroe (1979) identified 3 species, while Coulter and Rose (1900) identified 13 and Heywood (1971) and Tutin (1980) recognized 20 species [7,8,9,10]. Spalik and Downie (2001) revealed the polyphyly in *Anthriscus* based on nuclear ribosomal DNA internal transcribed spacer (ITS) sequences analyses [11]. Tekin and Civelek (2017) recognize both specific and subspecific variability [12]. According to POWO (2023), 14 species are currently accepted worldwide [13].

In Europe, Cannon (1968) distinguished four closely related species (*Anthriscus fumarioides* (Waldst. & Kit.) Sprengel, *A. nemorosa* (M. Bieb.) Sprengel, *A. nitida* (Wahlenb.) Garcke, and *A. sylvestris*), by the characteristics of the leaves and fruits [14]. The geographical and ecological differences between these species were marked by Hruška (1982) [15]. Some authors considered the variability of these taxa at the species level [16], while others have considered it at the subspecies level of *A. sylvestris* [5]. According to Euro+Med (2023), ten species of *Anthriscus* occur in Europe [17]. The morphologically similar *A. nitida* differs from *A. sylvestris* by growing in cold, shaded mountain valleys and by producing the sesquiterpene lactone grilactone [18]. *A. cerefolium* (L.) Hoffm., the species to which the etymology of the genus name “*Anthriscus*” is probably due, was known by the Greeks as a spice and is believed to have been part of the ladanum formulation along with *Cistus* species [4,19].

The primary habitat of *A. sylvestris* is typically alder riparian woods, but it is also found in disturbed wet habitats and along moist edges. It can establish dense colonies in various environments such as meadows, hay fields, hedgerows, road verges, ditches, and stream banks. Additionally, it is occasionally observed in wasteland areas, pastures, or open woodlands [2]. *A. sylvestris* thrives in soils ranging from moist to mesic, with an optimal pH level between 6.2 and 7.0 [20]. Both within its natural range and in areas where it has been introduced, it exhibits characteristics of a noxious weed in perennial forage crops and pastures and is now spreading in nature reserves, outcompeting other species [21]. The tendency to form dense monospecific populations and the presence of extensive bare soil patches surrounding the taproots indicate a potential allelopathic effect that hinders the establishment of other species [21].

The biological form of this species may vary depending on habitat conditions; it behaves as an annual, a biennial, or short-lived perennial in cold zones [7,22], or as a polycarpic perennial in subtropical ones [23].

The most common chromosome number reported for this species is 2*n* = 16 [15], but in the literature are also known values of 2*n* = 18 from Russian material [24] and 2*n* = 24 from the Himalayan region [25].

*A. sylvestris* is commonly known as wild chervil, and despite its invasive nature, interest in this plant has grown due to its beneficial aspects [4]. Numerous reports highlight its medicinal uses in traditional medicine, a topic to be further elaborated in the following chapter. Furthermore, *A. sylvestris* serves as a valuable source of lignan compounds, gaining significant attention in the fields of pharmacy and nutrition since the discovery of the aryltetralin lignan podophyllotoxin [26]. Podophyllotoxin serves as a key starting compound for the synthesis of etoposide and teniposide, effective anticancer drugs employed in the treatment of various leukemia and solid tumors [26,27].

Rhizomes of two Berberidaceae, *Podophyllum hexandrum* Royle (Indian, Himalayan) and *Podophyllum peltatum* L. (American), are used for isolating podophyllotoxin, with the Indian species being the primary source due to its higher content (4% of PT on dry weight) [26,28]. Unfortunately, the overexploitation of *P. hexandrum* has led to its designation as an endangered species. Recognizing the limited availability of the plant, significant efforts have been directed toward exploring alternative sources for lignan production [28,29].

In the biosynthetic pathway, podophyllotoxin is synthesized directly from deoxypodophyllotoxin [26]. Given that deoxypodophyllotoxin is one of the main lignans in *A. sylvestris*, it positions the plant as an excellent source for the production of podophyllotoxin [26]. Moreover, *A. sylvestris* is rich in diverse lignan compounds, possessing various biological activities, with cytotoxicity being one of the most intriguing and extensively studied. In light of the numerous side effects associated with current anticancer therapies, lignans from *A. sylvestris* emerge as potential novel anticancer drugs with enhanced selectivity and, consequently, reduced side effects [4].

Lignans, as plant secondary metabolites, can be found in various plant parts, including roots, rhizomes, stems, leaves, flowers, seeds, fruits, heartwood, bark, and resins. They predominantly exist in free form but can also be present as glycosides. In many plant species, they have a protective role against pathogens and predators, given their potent antimicrobial, antifungal, insecticidal, and other properties [30,31].

Additionally, lignans are present in mammals, either originating from the diet or as byproducts of gut microbiota action. Enterolactone and enterodiol, products of gut microbiota acting on matairesinol and secoisolariciresinol (lignans found in vegetables and fruits), are among the most recognized [30]. Some studies suggest that enterolactone is important for breast cancer prevention [32,33]. Elevated serum enterolactone levels have been associated with a reduced risk of breast cancer in both premenopausal and postmenopausal women [33]; however, the findings are still conflicting [34]. Vanharanta et al. propose a significant correlation between increased serum enterolactone concentration and a decreased risk of coronary heart disease and cardiovascular disease, as observed in a population-based study involving middle-aged Finnish men [35].

Several studies have documented the varied pharmacological activities exhibited by lignans, including but not limited to anticancer and antiviral properties, anti-inflammatory effects, and phytoestrogenic activity, underscoring the significant potential of these compounds. However, the exploration of *A. sylvestris* lignans has predominantly focused on the principal compounds, neglecting numerous lesser-abundant lignans that may possess substantial pharmacological relevance. Consequently, this paper aims to comprehensively outline all available biological activities of *A. sylvestris* lignans, thereby contributing to a more expansive understanding of the plant’s potential and providing inspiration for further research in this domain.

All the references cited in this review were obtained through thorough searches using Scopus, Science Direct, PubMed, and SciFinder-n databases. The search was performed within article titles, abstracts, and keywords using “*Anthriscus sylvestris*” as the main search term. Additionally, options such as “Cited by” and “Similar articles” were employed to identify additional relevant publications, along with references cited within the pertinent papers. SciFinder-n was additionally utilized to obtain data on the bioactivities of specific lignans.

### 1.1. Tradicional Medicine

The most investigated *A. sylvestris* lignans, podophyllotoxin and deoxypodophyllotoxin, are also known as “podophyllum lignans” since they were first found in *Podophyllum* species, as constituents of podophyllin resin. Podophyllotoxin was first isolated in 1880 by Podwyssotzki and DPT by Noguchi et al. in 1940 [36,37].

Podophyllin even became an official drug for a short time, in the U. S. Pharmacopoeia, in 1863. Besides *Podophyllum* species and *A. sylvestris* (family Umbelliferae), there are many other sources of lignans. *Podophyllum* lignans were also found in many Juniperus species and other members of the family Pinaceae, in two *Hernandia* species (family Hernandiaceae), in Cupressaceae, in *Linum* species, etc. [38,39].

*A. sylvestris* root was used as a crude drug “Zengo” in Japan. In China, under the name of “E Shen“, it was used as hematinic or tonic [40]. Dried roots have been used as an antipyretic, analgesic, antitussive, diuretic, and cough remedy in Korean traditional medicine [41,42], while a water extract from the flowers was used as a diuretic and tonic in Serbia [43].

Centuries ago, *A. sylvestris* was an ingredient in a cancer remedy described in the Leech Book of Bald, a medical book from preconquest England dating back to 900–950 A.D. In more recent history, an extract derived from this plant has demonstrated effective use in treating cancerous conditions [39]. In Gloucestershire, the Isle of Man, and Ireland, wild parsley was used as a remedy for kidney or bladder stones or gravel, although there is insufficient evidence to confidently attribute all records of wild parsley to *A. sylvestris* [44]. In Lesotho, it was used as a refreshing bath in the form of extracted lotion, while in Russia it was used as an abortifacient and remedy in childbirth. The powdered plant was used for dressing wounds in Europe [21]. The aerial part has been used in treating headaches in Tunisia [45]. In India, indigenous communities still utilize the plant to treat rheumatism and various other inflammatory conditions [21].

### 1.2. Official Medicine

While the plant itself does not have a direct application in official medicine, certain compounds and their semisynthetic derivatives have found practical use.

In pharmacotherapy, podophyllotoxin has various applications. In the form of a solution or cream, it is used topically as a home therapy for condyloma [46]. It represents a starting compound in the synthesis of three clinically applied anticancer drugs—etoposide, etopophos, and teniposide [29]. Clinical studies evaluated the activity of CPH 82 (Reumacon) as an antirheumatic drug containing two podophyllotoxin glucosides (podophyllotoxin-4,6-*O*-benzylidene-β-d-glucopyranoside and 4′-*O*-demethyl podophyllotoxin-4,6-*O*-benzylidene-β-d-glucopyranoside); the effect was comparable to methotrexate [47].

### 1.3. Nutritional Use

In Japan, *A. sylvestris* root was used as food. After being soaked in water, the root was crushed and pulverized, and used as food. Young aerial parts of plant were also sometimes used as food [40]. In Serbia, powdered wild parsley has been employed as a spice in salad dressings, yet it is not utilized as a functional food or in food technology applications [43]. In Turkey, particularly in the East Anatolian provinces, cheese infused with herbs is produced using plants gathered before flowering. Among the various plants included, fresh leaves and stems of *A. sylvestris* are also utilized. [48] In Britain, it is used as a pot herb [21].

## 2. Composition

### 2.1. Root Composition

A variety of lignans are known to accumulate in the roots of *A. sylvestris*, with the primary ones including deoxypodophyllotoxin, yatein, and anhydropodorhizol (nemerosin) [26,29]. Orčić et al. reported that the most abundant root components included deoxypodophyllotoxin (2.0–42.8 mg/g), nemerosin (2.0–23.4 mg/g), and yatein (1.1–18.5 mg/g), as well as podophyllotoxone (0.7–20.5 mg/g), guaiadequiol (0.8–8.3 mg/g), and dimethylmatairesinol (0.1–5.2 mg/g) [49]. Variations in the concentration of these main compounds have been observed among samples grown in different locations [26]. Deoxypodophyllotoxin, also referred to as anthricin, was initially isolated from *A. sylvestris* roots by Noguchi and Kawanami in 1940 [37]. Additionally, Kozawa et al. isolated anthricin, isoanthricin, and 2-(3′,4′,5′-trimethoxybenzyl)-3-(3′,4′-methylenedioxybenzyl)butyrolactone from the plant roots [50]. Other lignans such as nemerosin, (−)-deoxypodorhizone (yatein), and deoxypodophyllotoxin were isolated from the chloroform-soluble fraction [51]. Lim et al. were the first who identified angeloyl podophyllotoxin in the hexane fraction of the roots, alongside deoxypodophyllotoxin, while the chloroform fraction yielded morelensin and bursehernin [52].

Moreover, Koulman et al. identified 12 additional lignans in the roots, 6 of which had not been previously documented in *A. sylvestris*. These included arctigenin, dimethylmatairesinol, dimethylthujaplicatin, podophyllotoxin, 7-hydroxyyatein, and 7-hydroxyanhydropodorhizol [29]. Suzuki et al. identified yatein, secoisolariciresinol, nemerosin, and deoxypodophyllotoxin through GS–MS analysis, with secoisolariciresinol being reported for the first time in *A. sylvestris* [53]. Furthermore, Hedrawati et al. isolated nine lignans from the methanolic extract of *A. sylvestris*, including α-peltatin, β-peltatin, isopicropodophyllone, podophyllotoxone, and β-peltatin A methyl ether, five of which were isolated for the first time from this species [54].

Seeger et al. employed supercritical carbon dioxide extraction to isolate deoxypodophyllotoxin and anhydropodorhizol as the main lignan peaks, along with other lignans such as isopicropodophyllone, podophyllotoxone, yatein, and angeloyl podophyllotoxin [55].

In addition to lignans, various other compounds have been identified in *A. sylvestris* roots, including acyloxycarboxylic acid, phenylpropanoid esters, phenylpropanoids [50,54,56,57,58,59], terpenes [57,60], polyynes (polyacetylenes) [51,52,61], phytosterols, and fatty acids [57]. Phytochemical investigations have revealed the presence of anthriscusin, crocatone, and (*Z*)-2-angeloyloxymethyl-2-butenoic acid in the hexane root extract [56], while essential oil analysis showed a rich composition of terpenes such as α-pinene, β-myrcene, d-limonene, *p*-cymene, and sesquiterpenes [57,60]. Furthermore, various phytosterols and fatty acids were detected in the methanolic extract [57], highlighting the diverse chemical composition of *A. sylvestris* roots.

### 2.2. Aerial Parts Composition

Currently, there is limited information available on the chemical composition of the aerial parts of *A. sylvestris*. Phytochemical analysis conducted on these parts resulted in the isolation of two known lignans: deoxypodophyllotoxin and nemerosin [62]. Additionally, GS–MS analysis identified lariciresinol, matairesinol, hinokinin, pluviatolide and small amounts of bursehernin, with lariciresinol, matairesinol, and pluviatolide being reported for the first time in this species [53]. Recently, Orčić et al. provided insights into the lignan profile of the aerial components, highlighting higher proportions of aryltetralins like deoxypodophyllotoxin (consistently the most abundant lignan), podophyllotoxin, and acetylpodophyllotoxin, along with lower contributions of dibenzylbutyrolactones such as yatein [49].

In addition to lignans, the aerial parts contained various compounds, including phytosterol stigmasterol and several terpenes such as *o*-cresol, *p*-cresol, *p*-cumene, eugenol, and pentacyclic terpene quinovic acid, as identified by Milovanovic et al. [63]. Furthermore, the primary flavonoids identified in these parts were quercetin, apigenin, and rutin as the main quercetin glycoside [64]. Phenylpropanoids, phenylpropanoid esters, flavonoid luteolin 7-*O*-glucoside, and chlorogenic acid were also identified in aerial parts according to findings by Dall’Acqua et al. [62].

In the flowers, Ikeda et al. identified deoxypodophyllotoxin, morelensin, yatein, and (−)-hinokinin as lignan compounds [65]. Janković et al. conducted a quantitative analysis of ten lignans in the fruits of *A. sylvestris*, with the three most prevalent lignans being deoxypodophyllotoxin, yatein, and dimethylmatairesinol. Additionally, smaller quantities of podophyllotoxin, picropodophyllotoxone, guayadequiol, nemerosin, isochaihulactone, kaerophyllin, and isokaerophyllin were reported [66]. Notably, two compounds previously unreported in *A. sylvestris*, namely 7-*O*-hexosylpodophyllotoxin and 7-*O*-coniferylpodophyllotoxin, were tentatively identified [67].

Moreover, various terpenes, phytosterols, flavones, hydroxycinnamic acid, phenylpropanoids, phenylpropanoid esters, and polyacetylene were found [65,68]. GC–MS analysis of volatile compounds showed that the distinct floral fragrance stems from the ratio of monoterpenes with α- and β-pinene, *cis*- and *trans*-β-ocimene, limonene, sabinene, and myrcene as dominant compounds. The predominant volatile compound was (−)-sabinene, followed by myrcene and α-pinene [69].

Regarding the leaves, deoxypodophyllotoxin was the only reported lignan [68], followed by several terpenes and two phytosterols identified by Kurihara and Kikuchi and Bos et al. [60,68]. Hydrodistillation of fresh leaves yielded β-phellandrene as the main volatile compound. The main components were also present in dichloromethane extracts of the same plant material, but in significantly smaller amounts [60].

Despite containing lower amounts of deoxypodophyllotoxin compared to the roots, the aerial parts can be considered an alternative and renewable source of this lignan and also a promising component of functional foods [49,62].

## 3. Lignan Structure

Lignans, classified as plant secondary metabolites, encompass classical lignans, which are phenylpropanoid dimers, ββ-linked (C8-C8′) dimers of coniferyl alcohol, along with neolignans coupled by alternative C-C bonds, although the latter is not found in *A. sylvestris* [26,38,70]. These compounds originate from the phenylpropanoid biosynthesis route via the shikimic acid pathway and exhibit significant variation in oxidation level, substitution pattern, and the chemical structure of their fundamental carbon framework [71,72].

Lignans are categorized into eight subgroups based on how oxygen is integrated into the skeleton and the cyclization pattern: furofuran, furan, dibenzylbutane, dibenzylbutyrolactone, aryltetralin, arylnaphthalene, dibenzocyclooctadiene, and dibenzylbutyrolactol. Within each subgroup, lignans exhibit considerable variations in the oxidation levels of both the aromatic rings and propyl side chains [72]. In *A. sylvestris*, dibenzylbutyrolactone and aryltetralin lignans are the most prevalent [73].

Regarding structure numbering and nomenclature, different rules can be found in the literature. Previous numbering, and the most accepted according to Sackett et al., gave priority to the hydroxyl carbon (C7). Some prioritize the pendant ring, while Ayres and Loyke combined accepted rules with numbering based on the biosynthetic origin of lignans [74].

The nomenclature of podophyllotoxin-like compounds can also be diverse and confusing. Changes in chirality are labeled by prefixes attached to “-podophyllotoxin”, with some exceptions—in picropodophyllin “tox” is removed because of this compound’s biological inactivity. The distinction between the “normal” and “epi” series lies in the arrangement at C7, the difference between the “toxin” and “picro” isomers is attributed to the configuration at C8′, while C7′ isomers are labeled with “iso”. Sometimes C7′ chirality is described by D and L, where L stands for natural podophyllotoxin [38,39].

The term “iso” can be unclear because, in racemic isopodophyllotoxin, only the enantiomer with the natural C7′ configuration (thus, reversed configurations at C8′, C8, and C7) displays activity. In this context, “iso” denotes a reversal at C8′, C8, and C7 rather than at C7′ [38].

Dewick and Jackson, 1981 proposed a nomenclature, based on the α,β convention for podophyllotoxin substituents [75]. A simplified version involved naming spatial variations from the reference compound, podophyllotoxin. In that way, epipodophyllotoxin would be 7β-podophyllotoxin, picropodophyllotoxin would be 8′β-podophyllotoxin, isopodophyllotoxin would be 8′β, 8α, 7β-podophyllotoxin, etc. [38].

The subsequent discussion will delve into the brief biosynthesis of lignans, focusing particularly on those identified in *A. sylvestris*. Detailed structures and classifications will be illustrated in the provided figures.

The initiation of lignan synthesis begins with the radical coupling of two (*E*)-coniferyl alcohols, yielding pinoresinol. The next steps follow conversion from pinoresinol to lariciresinol to secoisolariciresinol, resulting eventually in dibenzylbutane matairesinol, the fundamental structure of classical lignans [53,70,76].

In the biosynthesis of lignans in *A. sylvestris*, matairesinol acts as a key precursor. After matairesinol, the process involves initiating steps such as forming a methylenedioxy bridge, methylating the p-hydroxy group, or both. One pathway results in the production of yatein via thujaplicatin, 5-methylthujaplicatin, and 4,5-dimethylthujaplicatin, while another pathway yields bursehernin through pluviatolide, both being dibenzylbutyrolactones. The conversion of matairesinol to yatein involves several steps: first, 5′-hydroxylation yielding thujaplicatin, followed by dual methylation at the 4′-OH and 5′-OH positions, resulting in 4,5-dimethylthujaplicatin. Finally, methylenedioxy bridge formation at positions 4 and 5 of the B ring occurs, forming ring A and producing yatein. The synthesis of bursehernin involves methylenedioxy bridge formation followed by methylation at the 4′ position of matairesinol [77,78]. The structures of all dibenzylbutyrolactone lignans found in *A. sylvestris* are shown in Table 1.

Structural diversity of dibenzylbutyrolactone lignans arises from multiple factors such as diverse substitutions on aromatic rings (methoxy, hydroxyl, or methylenedioxy), hydroxylation at the C8′, desaturation of C7-C8, C7′-C8′, or C8-C8′ bonds, and the presence of chiral centers at C8 and C8′ [70]. In addition to the dibenzylbutyrolactones shown in Table 1, *A. sylvestris* also contains various hydroxy- and oxo-dibenzylbutyrolactones, as well as unsaturated dibenzylbutyrolactones, detailed in Table 2 and Table 3. These lignans were primarily identified in *A. sylvestris* by our research group [73].

Aryltetralins—2,7′-cyclolignans with the dioxymethylene bridge on the B-ring, forming ring A, and the di- or trimethoxyphenyl functionality on the E-ring are further synthesized from yatein [29,81]. The oxidative cyclization of yatein produces an intermediate known as quinone methide, which can further cyclize to form deoxypodophyllotoxin. Additionally, the pivotal role of this quinone methide could explain the presence of podorhizol and anhydropodorhizol. These two compounds may arise from the quinone methide through processes such as the addition of water or the loss of a proton, respectively [81].

Deoxypodophyllotoxin, in turn, yields podophyllotoxin, which is then oxidized to podophyllotoxone. A comparable process is suggested for the analogous 4′-*O*-demethyl derivatives. Despite the ready conversion of 4′-*O*-demethyldeoxypodophyllotoxin into 4′-*O*-demethylpodophyllotoxin, neither compound is converted into lignans belonging to the 4′-methyl series, such as podophyllotoxin [81,82]. Transformation of deoxypodophyllotoxin to β-peltatin is also reported [72]. The structures of all aryltetralin lignans found in *A. sylvestris* are shown in Table 4.

In the literature, discrepancies emerge regarding the identification of deoxypicropodophyllotoxin and isoanthricin. Both Jeong et al. and Chen et al. reported deoxypicropodophyllotoxin, with Chen identifying the lactone hydrogen as characteristic of the picro series (R^2^ = αH and R^3^ = αH), whereas Jeong assigned R^2^ = βH and R^3^ = αH for the same compound. Since Jeong also accurately labeled another picro lignan, picropodophyllotoxin, we consider this labeling inconsistency an inadvertent error [42,90].

Furthermore, Kozawa reported isoanthricin, consistent with Chen’s findings [50,90]. However, upon comparing the proton NMR spectra provided by Kozawa with those of isoanthricin and deoxypicropodophyllotoxin reported by Chen, discrepancies emerged. Despite the low resolution of the spectra in Kozawa’s study, the comparison implies that the compound is more likely isoanthricin, albeit potentially impure.

In addition to the aryltetralin lignans listed in Table 4, *A. sylvestris* also harbors a variety of hydroxy- and oxo- aryltetralins, given in Table 5.

The subclasses aryltetralin and arylnaphthalene derive from dibenzylbutanes, arising from cyclization between C6-C3 units. Their main structural difference lies in the composition of the B ring, which can be either a cyclohexane ring in aryltetralins or a benzene ring in arylnaphtalenes [76]. The representative arylnaphthalene found in *A. sylvestris* is tetradehydropodophyllotoxin [73].

**Figure 1:**
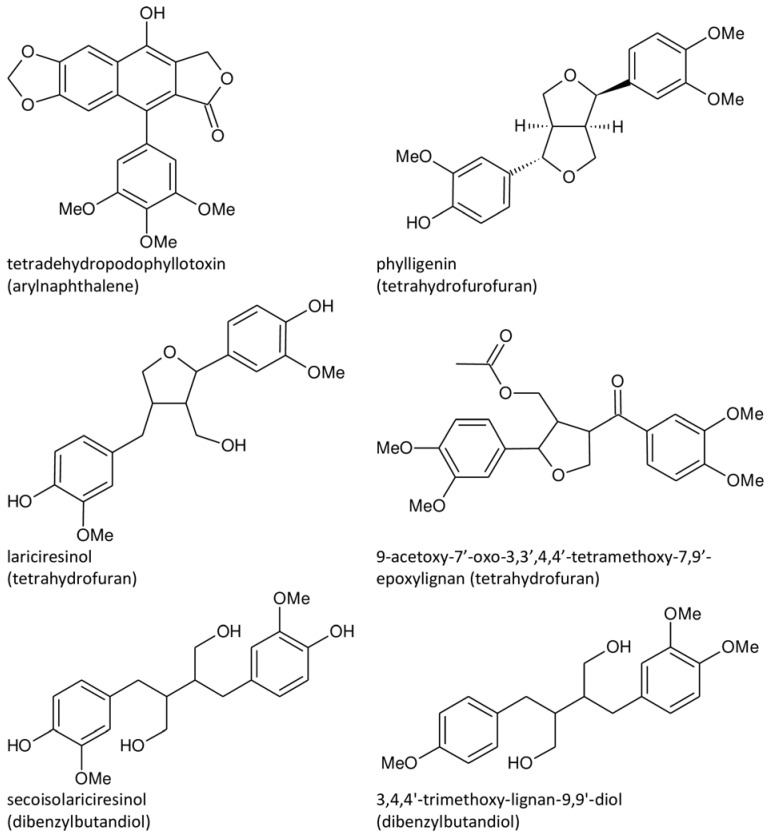
Other classes of lignans found in *A. sylvestris*.

Additionally, other classes are present in *A. sylvestris*, with representatives such as phylligenin (tetrahydrofurofuran) [26], lariciresinol [53] and 9-acetoxy-7′-oxo-3,3′,4,4′-tetramethoxy-7,9′-epoxylignan (tetrahydrofurans) [73], and secoisolariciresinol [53,73,77] and 3,4,4′-trimethoxy-lignan-9,9′-diol (dibenzylbutandiols) [73]. Their structures are given in Figure 1.

## 4. Biological Activity

The diverse structural variations found in lignans contribute to the numerous pharmacological activities reported within the lignan family. This chapter will focus on the biological activities of lignans discovered in *A. sylvestris*, encompassing anti-inflammatory, antiproliferative, antimicrobial, and antioxidant activities and toxicity towards animals, concerning potential mechanisms underlying these activities. A list of all abbreviations referenced in this review is provided in Abbreviation.

### 4.1. Anti-Inflamatory Activity

Anti-inflammatory activity, along with other properties, is predominantly studied using pure lignans rather than raw plant extracts. Although there are limited studies on plant extracts, this chapter will begin by referencing several such studies. Velescu et al. demonstrated that the extract from the aerial parts of *A. sylvestris* exhibited anti-inflammatory effects in rat models of induced inflammation. Considering that chlorogenic acid and luteolin 7-*O*-glucoside are the primary polyphenols in *A. sylvestris* aerial parts, each with inherent anti-inflammatory properties, it is reasonable to expect that the overall anti-inflammatory activity of the extracts will align with their concentrations [92]. However, since deoxypodophyllotoxin, along with a few other lignans, is also present in the aerial parts [62,65,68] and known for its broad biological activity, there is potential for synergistic effects when evaluating the activity of the plant extract.

Moreover, Lee et al. reported the aqueous extract of *A. sylvestris* leaves as a potential therapeutic agent for mitigating the progression of osteoarthritis (OA). This chondroprotective effect was investigated both in vitro using rat primary chondrocytes and in vivo in a rat model of destabilization of the medial meniscus (DMM) surgery-induced OA. The *A. sylvestris* leaves’ extract suppressed the expression of nitrite, iNOS, COX-2, interleukin-1β (IL-1β, proinflammatory cytokine and inducer of OA development)-induced inflammatory mediators, decreased IL-1β-induced degradation of aggrecan, collagen type II, and proteoglycan, and suppressed IL-1β-induced phosphorylation of MAPKs and NF-κB p65 subunit translocation to nucleus. Additionally, the extract inhibited in vivo induced cartilage destruction and proteoglycan loss [93]. The same research group also found that pretreatment with the same *A. sylvestris* leaves’ aqueous extract significantly suppressed the lipopolysaccharide (LPS)-induced secretion of nitric oxide (NO) and prostaglandin E2 in RAW264.7 cells, with no observed cytotoxic effects. These findings validated its anti-inflammatory activity, demonstrated by the suppression of NF-κB and MAPK pathways in vitro and by the inhibition of carrageenan-induced rat paw edema in vivo [94].

#### 4.1.1. Cyclooxigenase Inhibition

Deoxypodophyllotoxin (DPT) could provide a promising basis for novel nonsteroidal anti-inflammatory drugs (NSAIDs). NSAIDs alleviate pain and inflammation by blocking prostaglandin (PG) synthesis at the cyclooxygenase (COX) stage. The enzyme exists in two isoforms, COX-1 (constitutive) and COX-2 (inducible, typically associated with inflammation) [41]. However, commonly used NSAIDs inhibit the synthesis of PGs associated with both inflammatory and normal physiological processes, leading to significant side effects such as gastrointestinal ulcers and kidney dysfunction, limiting their safe and long-term use [41,95,96]. Therefore, finding more selective candidates is of high importance.

Anti-inflammatory activity examined on bone-marrow-derived mast cells (BMMC) resulted in a concentration-dependent inhibition of COX-1, with an IC50 value of 65.3 µmol/L, and COX-2, with an IC50 value of 1.89 µmol/L. Evaluation of a direct inhibition of COX-2 activity with a COX enzyme assay kit also showed concentration-dependent inhibition of COX-1 and COX-2 with IC50 values of 12.1 µmol/L and 0.01 µmol/L, respectively. The study demonstrated the potent COX-2 selective inhibitory activity of DPT [41].

In addition to cyclooxygenases, arachidonic acid metabolism can also be catalyzed by lipoxygenases, leading to the production of leukotrienes (LTs). The inhibition of 5-lipoxygenase (5-LOX) holds special interest due to the prominent proinflammatory role of leukotrienes and the approval of clinical treatments for asthma based on 5-lipoxygenase inhibitors and leukotriene receptor antagonists [97]. Deoxypodophyllotoxin (DPT) demonstrated a dose-dependent suppression of LTC4 biosynthesis in BMMC, with an IC50 value of 0.37 µmol/L, showing its potential in regulating immediate-type allergic reactions and its anti-inflammatory activity through dual inhibition of cyclooxygenase-2 and 5-lipoxygenase [41].

#### 4.1.2. Antiasthmatic Activity

In vivo antiasthmatic activity was evaluated on ovalbumin (OVA)-alum-induced asthmatic mice. Effects of DPT in the treatment of bronchial asthma in mice were assessed by quantifying the eosinophil count in the airway and analyzing the mRNA expression levels of eotaxin, Th2 cytokines, and the arginase isoform [84]. In the initial, sensitization stage, allergen-specific T lymphocytes become activated and responsible for the progression of allergic asthma upon re-exposure to the same allergens. Th2 lymphocytes are central to the development of allergic asthma. The airway inflammation is then characterized by Th2-produced cytokines including interleukins (IL) IL-3, IL-4, IL-13, IL-5, and type 2 innate lymphoid cells (ILC2s), the activation of mast cells, the infiltration and activation of eosinophils, and the increased production of immunoglobulin E (IgE) by B cells [98,99].

An elevated count of eosinophils in the airways, as observed in OVA-induced asthmatic mice, is associated with the severity of asthma highlighting their targeted elimination as a focus for asthma treatment [84,100]. Administering DPT before exposure significantly reduced eosinophil infiltration into the airways and lungs of OVA-challenged mice, in a dose-dependent manner. Additionally, experiments were conducted to ascertain whether DPT exerts its effects in reducing eosinophil numbers by inhibiting eotaxin and IL-5. Both eotaxin mRNA and IL-5 mRNA expression were inhibited by DPT in a dose-dependent manner [84].

Evaluation of DPT influence on Th2 cytokines expression revealed that the OVA-induced increase in mRNA expression of IL-4, IL-6, and IL-13 was prevented with DPT treatment in the mouse lung. Mice orally pretreated with DPT exhibited lower IL-4, IL-13, and IL-5 mRNA expression levels compared to control mice [84].

During allergic inflammation, Th2 cytokines induce the upregulation of arginases, resulting in increased production of proline and polyamines, and leading to airway remodeling. Increased polyamines influence cell proliferation and differentiation, while proline is the precursor of collagen and could affect allergen-induced fibrosis [101]. Additionally, elevated arginase activity leads to reduced nitric oxide synthesis, which may contribute to airway remodeling in chronic asthma, since NO normally inhibits airway smooth muscle (ASM) proliferation [102]. There are two arginase isoforms, cytosolic arginase I and mitochondrial arginase II, both expressed in the airways, but arginase I appears to be induced more strongly by IL-4 and IL-13 [84,101]. OVA-induced increase in arginase I expression was reduced by DPT, while arginase II mRNA was unaffected [84].

Many different inflammation processes, vasoconstriction, and tissue damage are affected by dysregulation of nitric oxide (NO). NO functions as a signaling molecule and exhibits an anti-inflammatory effect under normal physiological conditions but can become proinflammatory in abnormal situations with overproduction. Nitric oxide generated by inducible nitric oxide synthases (iNOS) serves as a significant mediator in both acute and chronic inflammation [103]. DPT inhibited lipopolysaccharide (LPS)-stimulated NO production in murine macrophage-like cells and affected the expression of iNOS. The exact mechanism of iNOS downregulation is not fully elucidated, but results suggest that DPT inhibits IκB degradation and NF-κB activation but does not influence MAPK pathway [85].

Kim et al. investigated the effect of *A. sylvestris* whole-plant extract on allergic lung inflammation both in vitro, using a Th2 polarization system, and in vivo, using an OVA-induced asthma mouse model. The extract reduced mucus secretion in airway epithelial cells and decreased inflammatory cell infiltration, eosinophilia, and Th2 cytokine levels in bronchoalveolar lavage fluid. Mice treated with the extract showed reduced expression of interleukin-6 and interferon regulatory factor (IRF) 4, along with decreased nitric oxide levels in the lungs of asthmatic mice and in stimulated RAW cells. These findings suggest that the extract suppresses Th2 cell activation by inhibiting IRF4 expression [104].

In another study, an extract of *A. sylvestris* was combined with Ramulus mori and Salvia plebeian. The researchers examined the effects of a fermented extract of these medicinal plants, which demonstrated antiasthmatic and antitussive properties without causing hepatotoxicity, thus offering the potential for enhancing respiratory well-being [105].

#### 4.1.3. Cardiovascular Effects

DPT demonstrated antiatherosclerotic and antiangiogenic potential. Namely, it was found that DPT inhibits the expression of matrix metalloproteinase MMP-9, as well as the enzymatic activities of both MMP-2 and MMP-9 in TNF-α-stimulated human aortic smooth muscle cells (HASMC). Furthermore, HASMC migration into the vascular wall was also inhibited [106]. On the one side, this reduces cellular damage that would promote atherogenesis. On the other side, it reduces angiogenesis, tumor vascularization, stromal remodeling, and metastasis [107].

Moreover, DPT induces cytoskeleton remodeling in human umbilical vein endothelial cells (HUVECs) via AMP-activated protein kinase (AMPK) stimulation, leading to the suppression of tumor vasculature in both in vivo and in vitro settings. The findings suggest that DPT selectively suppresses tumor vasculature by inducing cytoskeletal remodeling characterized by actin polymerization and microtubule depolymerization. Results indicate that microtubule depolymerization triggers activation of the RhoA/ROCK signaling pathway, in most cases responsible for tumor vasculature suppressing effect. Therefore, DPT holds potential as a therapeutic agent for targeting tumor vasculature in antimetastasis therapy [108].

#### 4.1.4. Hyperpigmentation Treatment

DPT shows potential as an efficient novel drug for treating hyperpigmentation caused via UV irradiation or by pigmented skin disorders [109]. Despite numerous pharmacological and cosmetic agents documented as melanogenesis inhibitors, only a limited number of these agents, due to toxicity or side effects, could be considered for the treatment of hyperpigmentation induced via UV irradiation or medical conditions like melasma [109,110]. Experiment on guinea pigs showed that 0.05% DPT, topically applied, removed UV-caused pigmentation after 14 days of treatment, while 2% hydroquinone (the most popular depigmenting drug, in clinical use since 1961) [110], reduced pigmentation only after 21 days of treatment. There was no side effect observed on the treated skin [109].

#### 4.1.5. Antiallergic Activity

DPT tested in a rat PCA (passive cutaneous anaphylaxis) assay, demonstrated dose-dependent inhibition of allergic reaction induced by IgE, surpassing the positive controls prednisolone and indomethacin. The intraperitoneal injection resulted in 30% inhibition, which was lower than indomethacin but slightly higher than prednisolone, under the same conditions. However, intravenous administration (0.25 to 1.0 mg/kg) exhibited strong dose-dependent inhibition, while oral administration (50 mg/kg) showed weaker efficacy, likely due to poor bioavailability. The exact mechanism of action is not clarified, but based on in vitro results, it is believed that in vivo inhibitory activity is related to the degranulation reaction. This study marks the first report demonstrating the antiallergic activity of deoxypodophyllotoxin in an in vivo animal model [83].

### 4.2. Antiproliferative Activity

Several studies have documented the antiproliferative effects of the methanolic/ethanolic extract of *A. sylvestris* root or the whole plant. Ikeda et al. discovered that only the chloroform-soluble fraction exhibited antiproliferative activity against MK-1, HeLa, and B16F10 cells [51]. Meanwhile, Cho et al. demonstrated potent cytotoxic effects against human gastric adenocarcinoma (AGS) cells with the n-hexane and methylene chloride fractions, and lower activity of the ethyl acetate and butanol fractions [89]. Moreover, the petroleum ether and chloroform fractions demonstrated significant inhibitory effects on HepG2 and HeLa cells, with IC50 values ranging from 18.25 to 45.66 μg/mL [90]. The main antiproliferative compound of the roots and aboveground parts of *A. sylvestris* was found to be deoxypodophyllotoxin [51]. Deoxypodophyllotoxin was also the dominant antiproliferative agent in *A. sylvestris* fruits [65]. Deoxypodophyllotoxin and angeloyl podophyllotoxin showed 100 times greater cytotoxicity towards human leukemia cells (K562), compared to etoposide and doxorubicin [52].

Muto et al. reported significant antiproliferative activity of DPT on human promyelocytic leukemia HL-60 cells, inducing apoptosis at very low nanomolar concentrations, while differentiation remained unaffected at similar levels. These findings also suggest that DPT and its derivatives exert antitumor effects primarily through cytotoxicity and apoptosis induction [111]. Antiproliferative activity of DPT and yatein (YAT) against human lung carcinoma and human melanoma cells is also reported [112].

Podophyllotoxin (PT) exhibits potent cytotoxic activity against diverse cancer cell lines. However, attempts to utilize PT as such have largely been unsuccessful, primarily due to complex side effects such as nausea, vomiting, and damage to normal tissues. Consequently, PT, in its current form, is not employed as a drug. Instead, it is utilized in the form of semisynthetic derivatives etoposide, teniposide, and etopophos. These derivatives are extensively employed for the treatment of various cancers, including lymphomas, acute leukemia, testicular cancer, small cell lung cancer, ovarian cancer, bladder cancer, brain cancer, and more [113].

PT and DPT exhibited significant inhibitory activity against L5178Y mouse leukemic cells in vitro [114]. In the case of PT, in vivo results also supported this, as confirmed with the P-388 lymphocytic leukemia screen in mice. Notably, none of the PT esters demonstrated higher activity compared to the parent molecule when tested at equivalent dosage levels [115].

Arctigenin (ATG) and matairesinol (MAT) demonstrated potent chemoprotective activity, with ATG exhibiting the highest chemoprevention index among the investigated compounds. Chemopreventive effects of ATG included the induction of phase II detoxification enzymes, shown by the induction of quinone reductase activity and apoptosis in in vitro cellular models (AGS cells) [116]. Furthermore, they demonstrated cytostatic efficacy against promyelocytic leukemia cells (HL-60) in vitro, exhibiting low cytotoxicity and selectivity towards normal immune-cell systems. Specifically, the cytotoxic effect on normal lymphocytes was nearly 11 and 150 times lower for AGT and MAT, respectively, compared to HL-60 cells, while for ET it was 2.8 times lower [117]. AGT and MAT’s potent antiproliferative activity against MH60 cells was again attributed to apoptosis rather than inhibition of IL-6 activity, as MH60 cells are IL-6-dependent [118]. In contrast, glucose-deprived PANC-1 cells treated with arctigenin underwent necrotic cell death, likely due to the inhibition of Akt activation, a critical process in cancer cell tolerance to glucose starvation. Arctigenin demonstrated preferential cytotoxicity against nutrient-deprived cells, with complete cell death observed at a concentration of 0.01 μg/mL within 24 h. Additionally, it showed efficacy against various pancreatic cancer cell lines (PANC-1, AsPC-1, PSN-1, BxPC-3, and the hepatoma cell line Alexander) and significantly suppressed PANC-1 tumor growth in nude mice [119]. Moreover, ATG has been reported to exhibit notable antitumor-promoting effects in mouse skin and pulmonary tumor two-stage carcinogenesis tests, along with immunomodulatory actions, including TNF-α and NO production and lymphocyte proliferation, and apoptosis induction in colon cancer cell lines [120,121,122].

Although apoptosis seems to be the primary mechanism of cell death induced by lignans, additional mechanisms are also present, including necroptosis. Interestingly, Ma et al. reported that DPT also triggers parthanatos. Their study on glioma cell lines and a mouse model of xenograft glioma showed that DPT induced glioma cell death in vitro and inhibited xenograft glioma growth in vivo. This was accompanied by parthanatos-related biochemical events, including hyperactivation of PARP-1, leading to cytoplasmic accumulation of PAR polymer, and nuclear translocation of AIF. Additionally, it was shown that deoxypodophyllotoxin induces parthanatos in glioma cells by promoting excessive ROS production [123].

Apart from compounds with well-studied mechanisms of action, several isolated compounds from *A. sylvestris* were assessed for their cytotoxicity (Table 6), displaying a range of activity from moderate to extremely potent.

#### 4.2.1. Antimitotic Activity

Microtubules have become recognized as crucial targets for anticancer treatments. They are composed mainly of the protein tubulin, which contains various binding sites for small-molecule drugs [132].

Microtubules are crucial for various cellular functions like mitosis, intracellular movements, cell motility, and intracellular transport, but especially for the separation of chromosomes during mitosis [38]. The dysregulation of mitosis, characterized by the loss of normal cell cycle controls in malignant cells, serves as a crucial target for anticancer agents, given that cancer cells often exhibit unscheduled and uncontrolled proliferation along with genomic instability [133].

Microtubules are ‘dynamic polymers’ composed of tubulin heterodimers formed from α and β tubulin monomers. Normally, they undergo dynamic assembly and disassembly, periods of growth, and shrinkage [132]. Regulatory mechanisms control this process, and small molecules from plants, such as colchicine, can disrupt microtubule regulation, acting as antimicrotubule agents and inducing depolymerization [38]. Ongoing interest persists in developing drugs that target the colchicine-binding site (CBS) of tubulin. Targeting the CBS offers several benefits, such as angiogenesis inhibition and the ability to overcome multidrug resistance (MDR) [132].

While microtubule-targeting agents (MTAs) have shown efficacy against different cancers, their usage in cancer therapy is constrained by significant side effects. Yet, the prospect of specifically blocking microtubule function during cancer cell division is appealing, with the challenge being to target essential mitotic regulators in cancer cells while sparing normal ones. The discovery of numerous overactive mitotic kinases (such as cyclin-dependent kinases, Cdks, or polo-like kinases, PLKs) in cancers has prompted the development of diverse antimitotic drugs [133].

Podophyllotoxin (PT) inhibits cell division by disrupting the assembly of microtubules in the mitotic spindle apparatus leading to cell cycle arrest at mitosis. Binding to the colchicine site of tubulin prevents the formation of mitotic-spindle microtubules and causes cell cycle arrest in the metaphase [27,71,113]. Compared to colchicine, the binding of podophyllotoxin was found to be faster, reversible, and less sensitive to temperature [38].

Deoxypodophyllotoxin (DPT), another aryltetralin lignan, also inhibits tubulin polymerization in various human cancer cells. It induces microtubule depolymerization in MCF-7 breast cancer cells, thereby eliciting its anticancer effects by inducing G2/M cell cycle arrest and caspase-dependent apoptosis [128,134]. Additionally, DPT exhibits a notably low resistance index (0.552), with studies demonstrating that it is not a substrate for P-glycoprotein, breast cancer resistance protein, or MDR-associated protein 2. This suggests a reduced likelihood of multidrug resistance (MDR) occurrence [134]. Studies showed the same antimicrotubule activity of DPT towards U-87 MG and SF126 glioblastoma cells 12 h after treatment with 30 nmol/L [129].

The dose-dependent tubulin polymerization inhibition by DPT was also observed on HeLa cervical cancer cells. G2/M phase arrest, determined via FACS (fluorescence-activated cell sorting) analysis, was detectable 12 h after the treatment. To elucidate the mechanism of DPT-induced G2/M cell cycle arrest, DPT’s impact on cyclin A and cyclin B1 expression was examined [86].

Cyclins and cyclin-dependent protein kinases (Cdks) are key regulators of cell cycle progression, implicated in the control of cell cycle progression, transcription, and neuronal function [135,136]. A-type cyclins accumulate during the S phase, forming complexes with Cdk1 and Cdk2. In the G2 phase, A-type cyclins undergo ubiquitin-mediated proteolysis, while B-type cyclins are actively synthesized. This leads to the binding of Cdk1 to B-type cyclins, a vital association for initiating mitosis, with a preference for the two main B-type cyclin isoforms, B1 and B2 [136]. The G2 phase continues until cell entry into mitosis, characterized by significant biosynthesis, primarily involving microtubule production that is essential for mitosis [137]. The transition from G2 to M phase is primarily controlled by the kinase activity of the Cdk1/cyclin B complex. Cdk activity is modulated by phosphorylation and dephosphorylation. Firstly, Wee1 and Myt1 kinases inhibit the activity by phosphorylating threonine and tyrosine residues; then, in the late G2 phase, Cdc25 phosphatases activate Cdk1 by dephosphorylating the same amino acid residues. Additionally, phosphorylation of the T-loop in the Cdk subunit via Cdk-activating kinase is necessary for the active Cdk–cyclin complex [136].

Treatment of HeLa cells with DPT reduced cyclin A expression and induced a rapid increase in cyclin B1 expression within 3 h of treatment. These changes suggest that DPT-induced G2/M arrest could be associated with alterations in cyclins A and B1 expression [86]. Furthermore, subsequent discoveries indicated that, besides the accumulation of cyclin B1, DPT treatment in HeLa triggers various processes, including the accumulation of polo-like kinase 1 (PLK1), along with the activation of Cdc25C and Cdk1. These findings suggest that DPT might induce cell cycle arrest at the G2/M phase by activating the Cdk1/cyclinB1 complex through Cdc25C, along with microtubule assembly inhibition [135]. Another study supported these findings, demonstrating that DPT suppressed the proliferation of gastric cancer cells (SGC-7901) and triggered G2/M cell cycle arrest, by leading to the accumulation of cyclin B1, Cdc2, and Cdc25C, while a decrease in the expression of Bcl-2 was observed. DPT also activated caspase-3 and PARP, indicating the involvement of caspase-mediated pathways in DPT-induced apoptosis. Animal experiments further revealed significant inhibition of tumor growth via DPT in a xenograft model of gastric cancer [138]. Additional studies reaffirmed DPT’s ability to induce G2/M cycle arrest by modulating the expression of cyclin B1, Cdc2, and Cdc25C proteins [128,129].

Wu et al. found that DPT effectively suppressed nonsmall-cell lung cancer (H460) cell proliferation in vitro and inhibited the growth of H460 xenografts in vivo, highlighting its potent antitumor activity against H460 cells. Furthermore, the study showed that DPT exerted a similar effect on both the drug-sensitive cancer cell line, H460, and the drug-resistant cell line, H460/Bcl-xL. DPT was observed to disrupt microtubules and arrest H460 cells at the G2/M phase. Notably, the study revealed, for the first time, that DPT induces necroptosis. The study marks the first documentation of necroptosis induction via a microtubule-targeting agent to overcome drug resistance in cancer therapy [139].

Interestingly, semisynthetic derivatives of podophyllotoxin primarily exert their effects by binding to the topoisomerase II-DNA complex; however, these differences are not always absolute. The mechanism of action for podophyllotoxin-like compounds, whether inhibiting tubulin polymerization or DNA topoisomerase II, may depend on concentration [38]. Crucially, these mechanisms are highly influenced by differences in structural characteristics of lignan compounds necessary for specific activity. In summary, for antimitotic activity, the stereochemical configuration of the C7, C8, and C8′ positions significantly contributes to tubulin binding, as does the presence of the lactone ring. Conversely, a bulky substituent at the C7 position shows no effect on microtubule assembly [140]. A more detailed discussion of the structure–activity relationship will follow in the next chapter.

#### 4.2.2. Topoisomerase II Inhibition

There are a few structural characteristics tightly connected to the inhibition of topoisomerase II. In summary, for this activity, the 4′-hydroxyl group was found to be necessary, as well as the (*S*) configuration of the C7 position (“epi” isomer) [140,141]. Despite podophyllotoxin’s antimitotic activity, the most known PT derivatives—etoposide (VP 16-213) and teniposide (VM-26)—act through topoisomerase II inhibition. Compounds similar to etoposide function by forming a complex involving a nucleic acid, drug, and enzyme. This complex induces breaks in both single- and double-stranded DNA, initiating a sequence of biochemical changes that ultimately result in cell death [130].

The administration of etoposide to murine mastocytoma cells (P-815) resulted in the inhibition of cell multiplication, noticeable as early as 1.5 h post-treatment, across both investigated concentrations (1 µg/mL and 10 µg/mL). Interesting observations included the persistence of DNA synthesis corresponding to the S and G2 phases, despite a significant inhibition of thymidine incorporation. This was also confirmed in cultures treated with VM 26 and in the relationship between uridine incorporation and RNA synthesis. The findings provided evidence for G2 cell cycle arrest while suggesting that influence on nucleoside uptake (as reflected in the inhibition of thymidine incorporation) and the cell block just before entering mitosis are independent effects [142]. Loike et al. confirmed this observation, discovering that several podophyllotoxin congeners (lacking the 4′-hydroxyl group) inhibit nucleoside uptake in HeLa cells but fail to demonstrate any effect on the fragmentation of DNA. Moreover, the nonglucoside derivative of etoposide and teniposide, such as 4′-demethylepipodophyllotoxin, demonstrated activity equivalent to glucosylated compounds in inducing DNA fragmentation and inhibiting nucleoside transport in HeLa cells. Notably, 4′-demethylepipodophyllotoxin also exhibited inhibition of microtubule assembly in vitro. This implies that cellular glucosidases might cleave the glycoside moiety, allowing 4′-demethylepipodophyllotoxin to exert its effects at distinct intracellular sites; however, there is currently no evidence confirming its accumulation in cells [141]. Additionally, compounds resembling podophyllotoxin act as antimitotic agents, arresting the cell cycle in the metaphase (G2/M), whereas etoposide and teniposide induce cell cycle arrest in the premitotic phase (S/G2) [27,71,142]. Huang et al. found that, aside from inhibiting cell growth, etoposide induced chromosomal aberrations during the G2 and S phases, implying the integrity of the glycoside moiety, as treated cells were arrested in the premitotic phase instead of the metaphase [143].

Given that certain lignans function as mitotic inhibitors while others act as topoisomerase II-DNA inhibitors, investigating the combination of chemotherapeutic drugs with diverse mechanisms of action would be valuable for further research.

#### 4.2.3. Caspase-3 Activity

The activation of the caspase cascade is a critical step in the apoptotic process, with caspase-3 and caspase-7 serving as the central effectors in the majority of apoptotic pathways [42,144].

DPT, angeloyl podophyllotoxin (APT), deoxypicropodophyllin (DPP), and picropodophyllotoxin (PPT) were tested for increasing activity of caspase-3 enzyme in human promyelocytic leukemia HL-60 cells [42]. Camptothecin (0.5 µmol/L), a potent inducer of apoptosis, served as the positive control. All investigated compounds demonstrated an increase in caspase-3 activity at concentrations of 0.001 µmol/L (for DPT) and 1 µmol/L (APT, DPP, and PPT). Notably, DPT exhibited the most potent increase in caspase-3 activity, inducing apoptosis even at very low concentrations. DNA fragmentation was observed with all lignan compounds [42].

Matsumoto et al. explored whether arctigenin’s effects on cell growth inhibition and cytotoxicity could be attributed to apoptosis. The study found that the induction of cell death by arctigenin was significantly impeded by the pan-caspase inhibitor, Z-VAD-FMK, suggesting caspase-mediated apoptotic cell death [118].

While several papers [42,86] imply that lignan molecules, especially the cyclohexane moiety of dibenzyl-γ-butyrolactones, directly activate caspase-3, this seems rather unlikely, since proteolytic activation by initiator caspase is a prerequisite [145]. Therefore, it is more likely that lignans induce apoptosis via upstream activation.

#### 4.2.4. Protein Kinase B (Akt) Inhibition

Activation of the protein kinase B (Akt, PKB) pathway is a common occurrence in numerous cancers, contributing to the inhibition of apoptosis and therapeutic resistance through various mechanisms. One of the primary roles of Akt, along with PI3K, is to promote cell survival and proliferation in response to extracellular signals. mTOR, a downstream component of the PI3K/Akt pathway and AMPK pathway, acts as a central regulator of cell growth and metabolism. Given this, major constituents of the Akt pathway, including PI3Ks, PDK1, Akt, and mTOR, are targets for cancer therapy research. Akt deactivation is a hallmark of both caspase-dependent and -independent cell death [146,147].

DPT shows potential as a breast cancer medication, inducing apoptosis in both estrogen-positive (MCF-7) and estrogen-negative (MDA-MB-231) breast cancer cell lines. It suppresses Akt/mTOR signaling in these cells by decreasing phosphorylated Akt and mTORC1 levels, crucial targets in breast cancer research [148], resulting in inhibited cell growth. Notably, inhibition of autophagy enhanced the DPT-induced apoptosis [88].

Park et al., discovered that DPT-induced apoptosis operates through the IGF1R/PI3K/Akt signaling pathway in human nonsmall lung cancer (A549) cells. This study marks the first report of IGF1R involvement in DPT-induced apoptosis in A549 cells [149]. The insulin-like growth factor 1 receptor (IGF1R) is a transmembrane tyrosine kinase receptor found abundantly in numerous cancers. Its downregulation induces significant apoptosis in cancer cells, making it a promising therapeutic target [150]. DPT inhibited IGF1R phosphorylation, consequently suppressing downstream PI3K and AKT phosphorylation, consistent with the inhibition of Akt/mTOR signaling observed as a mechanism of action [148,149].

Building on the evidence that DPT triggers apoptosis via the IGF1R/PI3K/Akt pathway in human lung cancer cells, Kwak et al. explored whether DPT operates similarly in esophageal squamous cell carcinoma (ESCC). They found that DPT directly inhibited the kinase activity of epidermal growth factor receptor (EGFR) and the phosphorylation of downstream signaling kinases, including AKT, GSK-3β, and ERK. DPT treatment not only suppressed ESCC cell viability and colony formation but also downregulated cyclin B1 and cdc2 expression, leading to G2/M phase cell cycle arrest, while upregulating p21 and p27 expression. These results suggest a therapeutic potential for DPT by inhibiting the EGFR-mediated AKT/ERK signaling pathway in ESCC [151].

The resistance to EGFR inhibitors presents a significant hurdle in targeted therapies for nonsmall-cell lung cancer (NSCLC). While tyrosine kinase inhibitors (TKIs) like gefitinib are the mainstay treatment for NSCLC patients with epidermal growth factor receptor (EGFR) amplification or sensitive mutations, the majority inevitably encounter disease progression as a result of acquired resistance to these agents. Resistance mechanisms often involve modifications in EGFR or its downstream pathways, underscoring the need for intensified clinical investigation into innovative agents that target prevalent resistance pathways, such as mesenchymal–epithelial transition (MET) expression [152,153,154].

Kim et al. examined the anticancer effects of DPT on HCC827GR cells resistant to gefitinib (EGFR-TKI) due to EGFR and MET regulation. Their findings revealed competitive ATP binding against EGFR and MET kinases by DPT, leading to reduced activities. Additionally, DPT treatment suppressed p-EGFR and p-MET expression, along with downstream proteins p-ErbB3, p-AKT, and p-ERK. It induced ROS generation, triggering endoplasmic reticulum stress and subsequent apoptosis through mitochondrial membrane potential loss and multicaspase activation [153]. Furthermore, Lee et al. investigated the effects of picropodophyllotoxin (PPT) on the same gefitinib-resistant cell line. Their results suggest a mechanism of action similar to that of DPT, wherein PPT binds to EGFR and MET within the ATP-binding pocket, inhibiting the activity of both kinases. This inhibition resulted in decreased phosphorylation of downstream proteins, AKT and ERK. Additionally, PPT induced G2/M cell cycle arrest, inhibited the growth of gefitinib-resistant NSCLC cells, and induced apoptosis by suppressing EGFR and MET activity [154]. These results suggest DPT’s and PPT’s potential as an adjuvant anticancer therapy targeting both EGFR and MET pathways [153,154].

In addition to the previously discussed mechanisms of the antiproliferative activity of lignans, this chapter concludes with a different application of *A. sylvestris* extract. Negut et al. conducted a study on the physicochemical and biological characteristics of magnetite nanoparticles functionalized with *A. sylvestris* extract (Fe_3_O_4_@AN). The in vitro toxicity assessment of Fe_3_O_4_@AN revealed significant cytotoxic effects against human adenocarcinoma HT-29 cells after prolonged exposure, although it was approximately three times less potent than the plant extract itself. The reported antitumor efficacy of the *A. sylvestris* extract is preserved in these coatings, with the developed nanostructured thin films ensuring controlled release for at least 3 days. Hence, these coatings offer practical solutions for developing natural and bioactive materials with multifunctional applications [155].

### 4.3. Antimicrobial Activity

#### 4.3.1. Antiviral Activity

Podophyllotoxin (PT) is employed as an antiviral treatment for condyloma acuminatum [46] caused by human papillomavirus (HPV) and is considered the most efficient remedy for venereal, perianal, and various common warts [71]. Its antiviral efficacy is linked to diverse mechanisms, such as the disruption of the cellular cytoskeleton and PT-induced necrosis in host cells, which hinders viral replication [71,156].

Saitoh et al. explored the interactions between podophyllotoxin (PT) and proteins associated with HPV 1a, a virus causing plantar warts. The study focused on the E2 protein, a functional component of HPV, which regulates viral transcription and DNA replication and consists of three domains: a transcriptional activator domain (AD), a hinge domain (HD) situated between two functional domains, and a DNA-binding domain (DBD) [157]. The HD region interactions encompass various cellular proteins, including the E7 protein (expressed in HPV-related carcinoma), nuclear matrix, and premRNA splicing factor. The investigation revealed that PT selectively interacts with the hinge domain of the E2 protein, without exhibiting affinity for other domains. Additionally, PT was found to inhibit the E2/E7 interaction by binding to the HD of the E2 protein, affecting the viral proliferation process, with etoposide showing a significantly weaker inhibitory effect than PT [156].

In addition to podophyllotoxin, other lignans—α-peltatin, β-peltatin, deoxypodophyllotoxin and picropodophyllotoxin—were also tested for antiviral activity. Based on suppression of cpe and reduction in infectious virus titers bioassay, PT exhibited the highest efficacy in inhibiting the replication of measles and herpes simplex type I viruses (HSV-1), with β-peltatin and deoxypodophyllotoxin showing minimal antiviral effects. Both α-peltatin and picropodophyllotoxin were inactive at the tested levels [158]. Additionally, PT showed low activity against HSV-2 [159]. Gordaliza et al. documented the antiviral effects of PT, DPT, and numerous PT derivatives at micromolar concentrations against herpes simplex type 1 (HSV-1) and vesicular stomatitis (VSV) viruses, which infect monkey kidney fibroblasts (CV-1) and hamster kidney fibroblasts (BHK), respectively [125].

Podophyllotoxin’s antiviral effect is likely due to the inhibition of microtubule assembly, with other mitotic poisons such as colchicine and vinca alkaloids also showing effects like reducing virus production or slowing down virus release from the infected cells [158].

At lower concentrations, podophyllotoxin selectively hinders the formation of unstable microtubules, like in the mitotic spindle, inducing cytostasis. Conversely, at higher concentrations, podophyllotoxin disrupts cellular microtubules, resulting in direct toxicity to resting cells. The antiviral efficacy of podophyllotoxin, observed at concentrations comparable to direct toxicity, suggests viral replication relies on microtubular structures crucial for the viability of resting cells. The authors propose that podophyllotoxin protects resting cells from HSV-1 infection by interfering with the microtubular transport system or impeding the formation of viral inclusion bodies [160].

PT and α-peltatin exhibited significant antiviral efficacy against murine cytomegalovirus (MCMV), with similar potency, reducing plaque formation by almost 50% at a concentration of 10 ng/mL. Nonetheless, neither compound influenced the formation of Sindbis virus plaques, possibly because RNA virus replication does not occur in the nucleus, eliminating the necessity for microtubular involvement in replication [160,161]. Antiviral activity against the Sindbis virus was evident only when the drug was administered at the same time as the virus, suggesting a potential inhibition of virus attachment or penetration, as pretreating the virus or post-treating infected cells had minimal to no effect [161,162].

Several studies have documented antiviral effects against human immunodeficiency virus (HIV), attributing them to mechanisms such as inhibition of topoisomerase II, viral integrase, or viral reverse transcriptase (RT) [162].

Arctigenin (ATG) and its glucoside, arctiin, exhibited potential in vitro antiviral activities against influenza A virus (IFV) but was less effective compared to oseltamivir. Arctiin metabolized into arctigenin in mice, with the latter significantly inhibiting virus replication when added immediately after viral infection, suggesting interference with early events of viral replication and suppression of progeny virus release. Oral coadministration of arctiin with oseltamivir in mice demonstrated in vivo synergistic therapeutic efficacy [163].

Arctigenin exhibits potent inhibition of HIV-1 replication in vitro [164,165]. The anti-HIV-1 efficacy could be attributed to its metabolism into certain O-demethylated congeners with HIV-1 integrase-inhibiting activity, or to its impact on an earlier stage in the viral life cycle [164]. Additionally, ATG strongly suppresses the expression of HIV-1 proteins P17 and P24 and significantly reduces reverse transcriptase activity [165,166]. Furthermore, ATG inhibits nuclear-matrix-associated DNA topoisomerase II activity, particularly in HIV-1-infected cells, suggesting a potential mechanism to prevent the increase in topoisomerase II activity associated with virus replication after HIV-1 infection [165].

Kim et al. found that ATG suppresses the PI3K/Akt pathway in HIV-1 Tat-expressing CHME5 cells [167]. Activation of phosphoinositide 3-kinase (PI3K) and its downstream effector Akt is crucial for the fibroblast transformation induced via various viral products. Furthermore, several human viruses, including HTLV, HPV, HCV, and HIV-1, also manipulate this pathway [167,168]. ATG demonstrated inhibition of phosphorylation in mTOR, GSK3b, and Bad. This suggests that arctigenin might broadly suppress downstream signals within the PI3K/Akt pathway [167].

The antiviral activity was found to be more selective for specific classes of lignans, emphasizing the importance of specific structural requirements [161].

The structural requirements for antiviral activity indicate that the C7 hydroxyl is dispensable, but the presence and the specific configuration of both rings, C and D, are required. Stereoisomers (picro- and epi-) and glucosides exhibit significantly lower activity compared to aglycones. The number and arrangement of phenolic hydroxyl groups are very important for the activity. [164] Additionally, replacing the lactone ring with another group leads to a notable decrease in activity [140,162,164].

#### 4.3.2. Antibacterial Activity

The antibacterial potential of *A. sylvestris* whole-plant extract was assessed by testing its n-hexane, methylene chloride (MC), ethyl acetate, and butanol fractions against *Escherichia coli*, *Staphylococcus aureus*, and *Helicobacter pylori*. Although the MC fraction showed slightly stronger activity against *E. coli* compared to other fractions, all fractions displayed similar activity against all tested bacteria using the disc agar diffusion method. Deoxypodophyllotoxin, isolated from the MC fraction, demonstrated activity against all the tested bacteria, especially *E. coli*. However, penicillin exhibited greater antibacterial efficacy (with a clear zone of 26 mm compared to 12 mm for DPT in the *E. coli* test) than both fractions and isolated DPT [89].

In addition to assessing its antitumor properties, Negut et al. examined the antimicrobial activity of *A. sylvestris* extract-functionalized magnetite (PLGA–Fe_3_O_4_@AN) nanoparticles. The antibacterial efficacy was evaluated against *Staphylococcus aureus* and *Escherichia coli*, while antifungal activity was evaluated toward *Candida albicans*. The results confirmed the antimicrobial effectiveness of the *A. sylvestris* extracts and demonstrated that the developed coating could facilitate sustained activity of this extract for at least 72 h [155].

### 4.4. Antioxidant Activity

With the rising significance of natural antioxidants in food technology, the evaluation of plants’ antioxidant potential became popular. Ethanolic extracts of aerial parts of *A. sylvestris* exhibited potent, concentration-dependent antioxidant activity. Rancimat analysis at 100 °C demonstrated that the antioxidant activity of the 70% ethanolic extract of *A. sylvestris* surpassed that of quercetin, apigenin, or a tocopherol mixture [64]. Dall’Acqua et al. assessed the same activity from methanolic extracts (MTE) of aerial parts, and for isolated compounds. DPPH testing of MTE resulted in an IC50 of 184 ± 10 µg/mL, while the highest antioxidant activity was observed in fraction F6, with an IC50 of 9 ± 2 µg/mL. From this fraction, luteolin 7-*O*-glucoside and chlorogenic acid were isolated. Antioxidant activity of isolated compounds pointed out chlorogenic acid as the most active (IC50 = 5.6 ± 0.1 µg/mL), compared to luteolin glucoside (IC50 = 15.5 ± 0.2 µg/mL) and deoxypodophyllotoxin as the least active (IC50 = 61.0 ± 2.4 µg/mL) [62].

Cho et al. reported the radical-scavenging potential of n-hexane, methylene chloride (MC), ethyl acetate, and butanol fractions from *A. sylvestris*. At a concentration of 100 µg/mL, all fractions demonstrated scavenging of over 85% of both DPPH and hydroxyl radicals, with the n-hexane and MC fractions being the most potent [89].

### 4.5. Other Activities

#### 4.5.1. Cytochrome P450 (CYP) Inhibition

Cytochrome P450 (CYP) enzymes, heme-thiolate monooxygenases, play a crucial role in the metabolism of a wide range of structurally diverse drugs and xenobiotics, where CYP3A4 is the main human metabolizing enzyme [169,170]. These enzymes are involved in phase I of xenobiotic metabolism and catalyze the oxidation, reduction, or hydrolysis of primarily lipophilic xenobiotics into more polar molecules, which then undergo biotransformation in phase II to the final excretion in phase III [169]. Since the impact of drug–drug interactions can be significant, understanding the CYP system plays an important role.

Julsing et al. compared the effects of five lignans (deoxypodophyllotoxin, epipodophyllotoxin, podophyllotoxin, demethylenedeoxypodophyllotoxin, and demethylenepodophyllotoxin) on CYP3A4 to examine the impact of the methylenedioxy group on the biotransformation process. Demethylenepodophyllotoxin, lacking the methylenedioxy moiety, did not induce inhibition, confirming that the inhibitory impact is associated with this group [170]. This can be explained by the oxidation of the methylenic carbon in methylenedioxyphenyl compounds to a carbene, subsequently interacting with the ferric form of CYP3A4 to form a stable heme-adduct complex [170,171]. Additionally, human CYP3A4 metabolizes deoxypodophyllotoxin into the stereoisomer epipodophyllotoxin as the sole metabolite, and the observed inhibition may be attributed to either competition for the enzyme or inhibition by the formed product [170].

Lee et al. explored the inhibitory effects of DPT on CYP enzyme activities and its mechanism in human liver microsomes (HLM) and human CYPs expressed in baculovirus-insect cells to assess the potential for drug–drug and/or herb–drug interactions involving DPT [172]. Cocktail probe assay resulted in inhibitory activities of DPT on HLM CYP2C9 and CYP3A4 in a concentration-dependent manner, with IC50 values of 6.3 and 9.2 µmol/L, respectively [172].

DPT’s inhibition of CYP3A4 was demonstrated to be competitive, aligning with a previous report, while the secondary plot for CYP2C9 in HLM exhibited a nonlinear pattern, suggesting a potential parabolic competitive inhibition, possibly influenced by products of DPT metabolism in HLM. These findings indicate the potential for interactions between DPT and drugs metabolized by CYP2C9 or CYP3A4, either as herb–drug or drug–drug interactions [172].

#### 4.5.2. Neurotoxic Effect

Xu et al. studied the neurotoxic effect of DPT on widely used rat dorsal root ganglion (DRG) neurons. In the study, voltage-gated sodium channels had been studied as principal molecular targets of DPT, and the possible synergistic actions of DOP on Ca^2+^ and membrane excitability had also been evaluated [173].

DPT inhibited both TTX-S (tetrodotoxin-sensitive) and TTX-R (tetrodotoxin-resistant) sodium currents in rat DRG neurons in a concentration-dependent manner, leading to a reduction in the number of action potentials (APs) in current clamp experiments. The suppression of sodium currents, which are crucial for action potential generation and excitability, may significantly contribute to the antitumor and anti-inflammatory activities of DPT [173]. Numerous pieces of evidence indicate de novo expression of voltage-gated sodium channels (VGSCs) in various human carcinomas, influencing metastasis-related cellular behaviors; this is consistently reflected in elevated tissue sodium levels in cancer compared to noncancer tissues. Considering that, inhibition of Na^+^ channels could be one of the anticancer activity mechanisms of DPT [174,175]. Moreover, the inhibition of Na^+^ channels in sensory neurons serves as a crucial mechanism for DPT to exert its analgesic functions.

Calcium ion (Ca^2+^) signaling holds a unique significance in the nervous system, serving as the principal second messenger to regulate neuronal and synaptic activities. In homeostasis, intracellular Ca^2+^ levels [Ca^2+^]_i_ are maintained at an appropriately low level [176,177].

DPT increased [Ca^2+^]_i_ in a concentration-dependent manner. The increase in [Ca^2+^]_i_ in a Ca^2+^-free environment indicates that DPT can induce internal Ca^2+^ release, while the substantially greater increase in [Ca^2+^]_i_ observed upon reintroducing Ca^2+^ into a Ca^2+^-free solution indicates DPT’s capacity to mobilize external Ca^2+^. These findings not only offer a foundation for the medical applications of DPT but also indicate its cytotoxic effect on the nervous system through the elevation of intracellular calcium in mammalian neurons [173].

#### 4.5.3. Protective Activity

Jang et al. identified arctigenin (ATG) as a potent neuroprotective agent against glutamate-induced toxicity, with a concentration range of 0.01–10 µmol/L. The highest protection was observed at 1 µmol/L, while the efficacy decreased with higher concentrations [178]. Moreover, ATG demonstrated notable hepatoprotective activity in primary cultures of rat hepatocytes damaged by carbon tetrachloride (CCl4), by maintaining the glutathione (GSH) levels. Moreover, Moritani et al. reported a cytotoxic effect on HepG2 cells attributed to ATG, which was detoxified by GSH [131]. Further investigation is required to understand how lignans protect hepatocytes through GSH preservation, as the mechanism seems to involve factors beyond antioxidative properties [179].

Additionally, DPT demonstrated potential in treating hepatic steatosis. Oral administration of DPT significantly suppressed fatty liver induced by a high-fat diet in mice. Analysis of the regulatory mechanism revealed that DPT inhibited the induction of SREBP-1c and the expression of lipogenic genes. DPT also activated AMPK, which is known to inhibit the expression of SREBP-1c in hepatocytes, suggesting that DPT modulates lipid metabolism through coordinated AMPK activation and SREBP-1c inhibition [180].

#### 4.5.4. Immunomodulatory Activity

Gordaliza et al. reported that several cyclolignans might suppress a T-cell-mediated immune response via a noncytotoxic mechanism of action. In vitro evaluation with mixed lymphocyte reaction placed α- and β-peltatin among the most active representatives [181].

In vivo administration of *A. sylvestris* extract to an (OVA)-specific TCR transgenic mouse model stimulated the production of proinflammatory cytokines via macrophages, resulting in the activation of IL17-producing innate immune cells like NKT cells and neutrophils, at the early time point and later, also inducing IFNγ- or IL17-producing adaptive immune cells such as Th1 and Th17 cells, while concurrently reducing Treg cell levels. Results suggested that *A. sylvestris* extracts could serve as promising therapeutics for treating Th2-associated immune diseases such as atopic dermatitis [182].

### 4.6. Toxicity towards Animals

Toxicity of deoxypodophyllotoxin and its stereoisomer, deoxypicropodophyllin, was tested on two fish species—Japanese rice fish (*Oryzias latipes*) and goldfish (*Carassius auratus*)—as well as brine shrimp (*Artemia salina*) and red worms (*Limnodrilus hoffmeisteri*). It was shown that DPT was toxic towards all investigated species, which was expected since phenylnaphtalide lignans have ichthyotoxic activity. DPT also showed phytogrowth-inhibitory activity towards two plant roots (*Medicago sativa* L. and *Brassica rapa* L.), at a concentration of 50 ppm. The picro isomer, deoxypicropodophyllin, showed no activity, attributed to the difference in stereochemistry between these compounds [183]. Similar to its ichthyotoxic activity, the phytogrowth-inhibitory effect of podophyllotoxin (PT) was found to be less potent than that of DPT [114].

Deoxypodophyllotoxin and β-peltatin A methyl ether demonstrated high insecticidal activity against several insect species. In contrast, their C2-epimers, deoxypicropodophyllin and β-peltatin B methyl ether, were not insecticidal, suggesting that stereospecificity has a significant influence on the mechanism of action [40,184]. Similar to DPT, PT also demonstrated insecticidal activity, although less potent overall, except in the case of *Epilachna sparsa orientalis* larvae, where it exhibited a much stronger effect than DPT [114]. Inamori et al. examined the mechanism of insecticidal activity of DPT as one of the most potent insecticidal agents. Same as Kozawa et al. (1982), the paper also showed that DTP has a delayed activity—symptoms of intoxication develop slowly, 24 h after ingestion [40,185]. Since most insecticides are rapidly acting neurotoxic agents, the DTP mechanism of action is likely different from the mechanism of neurotoxicants [185]. Inamori et al. continued to examine the exact mechanism by publishing histopathological studies of tissues of silkworm larvae. The study confirmed that previous observations (greatly decreased food ingestion and greatly decreased feces excretion) are related to the destructive action of DPT on epidermal cells [185,186]. Inamori et al. concluded that the mechanism of insecticidal activity involves severe damage to the epidermal cells which causes delayed symptoms after intoxication [186]. However, attention should be paid to the lignans’ high cytotoxicity [184].

## 5. Structure–Activity Relationship

Structure–activity relationship (SAR) studies play a crucial role in guiding the design and synthesis of novel derivatives with potential antitumor activity. To determine how structural changes influence the activity of lignans, many podophyllotoxin compounds and structural analogs have been investigated. Structure–activity studies showed that alterations of some regions of the molecule affect the biological activity more than others.

**Figure 2:**
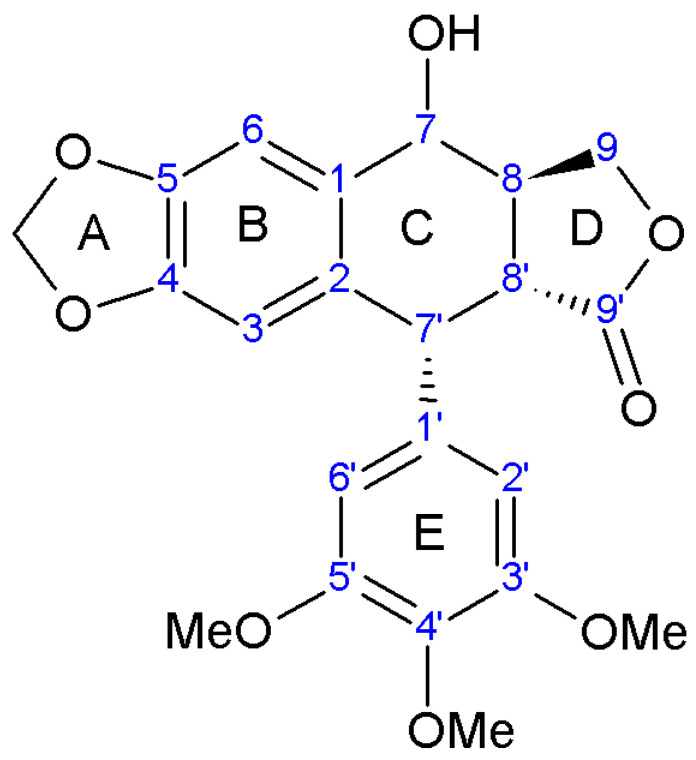
Structure of podophyllotoxin, with numbering (according to IUPAC nomenclature of lignans [187]) and ring labeling.

The main modifications have been focused on rings C, D, and E, (Figure 2) with comparatively fewer reports regarding rings A, B, and D. Among these, the C7 positions of ring C have been identified as producing more favorable derivatives [113]. These influences will be elaborately described below.

### 5.1. Ring A Modifications

Contrary to the previous belief that all the rings are necessary for podophyllotoxin activity, the current understanding is that only the A (dioxolane) and E (pendant phenyl) rings are essential [113]. The importance of the A ring was also confirmed by Terada et al., who stated that in podophyllotoxin compounds, ring A is required for inhibition of both targets, tubulin polymerization and topoisomerase II [188]. Additionally, the methylenedioxy ring of deoxypodophyllotoxin derivatives was found to be essential for the enhancement of cytotoxic activity [112]. Castro et al. investigated podophyllotoxin derivatives lacking the methylenedioxy group and/or with different functionalizations on the A ring, such as acetylation of the catechol groups. The tested derivatives exhibited cytotoxicity levels significantly lower—two to three orders of magnitude—than those of the parent compounds podophyllotoxin and deoxypodophyllotoxin. Nevertheless, the cytotoxicity remained at the micromolar level [189]. On the other hand, Gordaliza et al. reported that the removal of the methylenedioxy group through demethylenation appears to be a suitable modification to maintain, or possibly enhance, the immunosuppressive activity of cyclolignans [190].

### 5.2. Ring B Modifications

Some of the best-known B-ring variants of podophyllotoxin are natural products—α-peltatin, β-peltatin, β-peltatin methyl ether, and others, though not that many B-ring derivatives have been synthesized, compared to C- or E-ring derivatives. Replacing the OH-group with hydrogen at position C6 results in no significant reduction in antimitotic activity of α- and β-peltatin [27]. Derivatives with a hydroxyl group at the C6 position exhibit greater activity compared to those with a hydroxyl group at C7. However, in both cases, picro isomers, as well as glucosides, demonstrate no antimitotic activity [38]. Moving glycosidic moiety from C7 to aromatic C6 resulted in much lower topoisomerase II inhibitory activity and cytotoxicity, which is in agreement with the importance of axial-axial configuration across the C (cyclohexane) ring for antitopoisomerase activity [38,191]. Some specific substitutions at the C6 hydroxyl can enhance the activity. For example, α-peltatin methyl ether showed much higher cytotoxicity than α-peltatin, and since antitopoisomerase activity was low, it can be assumed that the activity is related to microtubule polymerization inhibition. Other α-peltatin C6 derivatives (acetate, succinate, phenylacetate, and carbobenzoxy esters) also showed high cytotoxicity and little or no inhibition of topoisomerase [191]. 6-methoxypodophyllotoxin (normally accumulated in Linum flavum) showed cytotoxicity comparable to podophyllotoxin [192].

### 5.3. Ring C Modifications

Under the influence of mild base catalysts, all podophyllotoxin compounds smoothly undergo epimerization at the 8′-position, leading to lignans of the picro series. However, these derivatives exhibit minimal to negligible cytotoxic activity [39]. The inactivity of the picro isomer was also confirmed by Inamori et al. who described spatial conformation of DPT and deoxypicropodophyllin. They found that the 8′–lactone carbonyl bond was quasi-equatorial in DPT, and quasi-axial in picro isomer. Also, the C-ring has the half-chair form in DPT and half-boat form in the picro isomer. It was not possible to conclude whether the differences in activity were due to bond orientation, or C ring shape [183].

SAR studies showed that, for the antitumor activity of podophyllotoxin, but also etoposide, *trans* conjugation between C and D (lactone) rings is necessary. Corresponding cis-hydroxy acid and *cis*-picro-lactone are more stable, but not biologically active in vitro. *Cis*-picro-lactone isomer can be produced under physiological conditions, but also in base conditions, such as treatment with ammonia, sodium carbonate, and sodium hydroxide [193].

Substitution of the 7β-O-glucosidic moiety of etoposide with several 7β-arylamino moieties results in more potent inhibitors of human DNA topoisomerase II, compared to etoposide [193]. The presence of the bulky groups at the C7 position enhances topoisomerase activity [113]. The toxicity and ability to damage tumors were notably lower for the lignan glycosides compared to their corresponding aglycones [39]. A comparison of etoposide and 4′-O-demethylepipodophyllotoxin also confirmed this, as the aglycone is a microtubule inhibitor [27].

The potential of lignans to damage tumors is significant only if they have identical configurations at C8, C8′, and C7′ as podophyllotoxin—*trans*-(7:8)-*trans*-(8:8′)-*cis*-(8′:7′) [39].

Investigation of podophyllotoxin and its derivatives, including cyclic ether, cyclic sulfide, and cyclic sulfone derivatives of podophyllotoxin and deoxypodophyllotoxin; epipodophyllotoxin; etoposide; teniposide; and picropodophyllotoxin, showed that C-ring substitution plays an important role in interactions with tubulin. The inhibitory activity is affected by the configuration, size, and chemical nature of substituents at C7 [27,194]. Epipodophyllotoxin showed lower activity compared to PT and DPT, consistent with Castro et al.’s observation that the epimerization of the hydroxyl group at C7 reduced cytotoxic potency [27,189]. Etoposide and teniposide (4′-*O*-demethylepipodophyllotoxin ethylidene-β-D-glucoside and thenylidene-β-D-glucoside, respectively) did not inhibit microtubule assembly, indicating a different mechanism of antitumor activity. The increasing hydrophilic nature of C7 substituents also lowers antimitotic activity [27,194].

Incorporating an aminoalkoxy group at position C7 enhances the inhibitory effect on topoisomerase II and cytotoxicity, resulting in the elimination of tubulin polymerization inhibition as a mechanism of activity. Other hydroxyalkoxy or alkoxy groups show less potential. As expected, all 7-alkoxy derivatives exhibited decreased or no tubulin polymerization inhibition at all [188].

Gordaliza et al. found that derivatives lacking a substitution at C7 exhibited greater effectiveness against neoplasms and demonstrated significantly stronger antiviral activity compared to derivatives with an oxygenated group at the same position. The presence of a 7α-hydroxyl group had minimal impact on activity, while 7β-hydroxyl or 7α- or 7β-methoxyl groups reduced it by 6–10 fold. Chlorine substitution had a markedly lesser effect [125,126]. The consistent correlation observed between antineoplastic and antiviral outcomes could suggest the presence of a shared mechanism of the activity of cyclolignans against both cells and viruses [126].

Additionally, derivatives with no substitution at C7 were also found to be potent immunomodulatory agents. For compounds with oxygenated substituents, potency decreases in the following order: α-OH > β-OH > OAc > CO [181].

Interestingly, antitumor activity in vivo showed that, despite the high inhibitory effect on tubulin polymerization, cytotoxic activity can be low. This means that a potent cytotoxic effect alone may not reliably indicate effective antitumor activity in vivo if it is linked to the inhibition of tubulin polymerization [188]. Cytotoxic activities of several 4′-O-demethylepipodophyllotoxin derivatives are given in a review paper by Srivastava et al. The derivatives, all modified at C7, included 7β-amino derivatives and their corresponding hydrochloride salts, 7β-nitroaniline derivatives, 7β-amido, and sulfonamido derivatives, acetamido and formamido derivatives, and sulfonamide derivatives with methyl, propyl, 3-chloropropyl, azidopropyl, and other substitutions [113]. Within each group, a select few derivatives demonstrated greater cytotoxic activity than etoposide. However, it is essential to note that achieving comparable results in in vivo tests is not guaranteed. This distinction between in vitro and in vivo test outcomes has also been highlighted by You et al. [127].

Saturation of the C ring seems to be necessary for antimitotic activity, as dehydropodophyllotoxin showed no antimicrotubule activity [27].

Configuration on C7 also influences the other lignan mechanism of action—DNA fragmentation. Compounds having a 4′-hydroxyl group and “epi” configuration of C7, were more active than the other isomer [141]. The greater activity of teniposide, compared to that of etoposide, suggests that the chemical nature of a C7 substituent also influences DNA fragmentation, but a glycoside moiety of these compounds is not necessary for activity, since aglycone derivatives are also active [141].

### 5.4. Ring D Modifications

Antineoplastic activity of podophyllotoxin-like compounds is linked to the lactone moiety, with differences between *trans*- and *cis*-lactones due to stability and molecular shape. *Trans*-lactones, being more strained and less stable, readily convert to the *cis*-8′-epimers in the presence of a base and even in neutral methanolic solutions and demonstrate a higher ability to form covalent bonds with the target biomolecule [125,126]. Additionally, while the four rings in *trans*-lactones are nearly coplanar, in the primary conformer of *cis*-isomers, the lactone adopts an almost perpendicular orientation in relation to the other three rings. Both factors, stability (reactivity) and conformation (accessibility), can account for the enhanced antineoplastic and antiviral activity of *trans*-lactones [125,126].

Configuration of lactone ring affects the inhibitory activity of tubulin polymerization. The *Cis* configuration of the D ring, as in picropodophyllotoxin, is almost inactive compared to the *trans* lactone ring as in podophyllotoxin [195]. Steric features of the D ring also influence the antimitotic activity of lignans [194].

The cytotoxic effect of the yatein (dibenzylbutyrolactone) analogues displays stereospecificity since (−) yatein showed high cytotoxic activity, but racemic yatein resulted as nearly inactive. This indicates that (+) yatein acts as an antagonist to the active (−) yatein at a receptor binding site, albeit with an unclear mechanism of action [112].

The lactone group in this ring is not necessary for antimitotic activity, since substitution of the carbonyl group by the methylene group resulted in no significant decrease in activity. On the other hand, the effects of substitution of lactone oxygen are significant and depend on the size of the replacing group. Decreasing activity is in correlation with the der Walls radius of the substituents—the higher the radius, the lower the antimitotic activity of the compounds, suggesting that the binding site in tubulin has specific steric requirements in the part where lactone oxygen interacts with tubulin [27,194]. Derivatives with an opened lactone ring have decreased activity or are inactive [38,125]. The immunomodulatory activity is also reduced by the opening of the lactone fragment [181]. Yet, if the opening of the lactone ring is accompanied by the formation of a fused isoxazole heterocycle, it appears to be a suitable modification to maintain or potentially enhance the immunosuppressive activity of cyclolignans, while decreasing the antineoplastic potential [130,190].

The presence of the D ring is, however, necessary for antimitotic activity, since derivatives missing the D ring, or modified so that the lactone ring is free to rotate (open C ring), are inactive [27].

### 5.5. Ring E Modifications

Investigation of several podophyllotoxin analogs indicated the necessity of quasi-axial (*trans*) positioning of the E ring (in relation to the C ring) for antimitotic activity [194].

The situation is different in picropodophyllotoxin. Picropodophyllotoxin has *cis*-lactone conformation, which allows free E ring rotation [194]. Brewer et al. found that the predominant conformation of picro isomer is equatorial [194]. This is in agreement with Loike et al., who found tetradehydropodophyllotoxin (with an aromatic C ring and an E ring coplanar with the rest of the molecule) inactive [27].

Since picropodophyllotoxin is a conformationally flexible molecule, the reduced activity could be explained through the presence of both conformations, the dominant one (equatorial) being inactive and the other (quasi-axial) active but present in the lower amount [194].

Rithner et al. found that in the stable conformation of podophyllotoxin, the pendant ring has restricted rotation which results in a preferred position where the E ring is essentially perpendicular to the rigid structure of the molecule [196]. On the other hand, Hu et al. suggested that free rotation of the E ring is necessary for DNA topoisomerase II inhibitory activity [193]. Chloro-derivatives in 2′-position (2′-chloroetoposide, 2′-chloro-7β-(arylamino)-4′-O-demethylpodophyllotoxins) showed no cytotoxic activity towards the examined cell line and exhibited significantly lower activity in a DNA topoisomerase II inhibition assay. By adding a chlorine atom in the 2′-position, rotation of the E ring is restricted because of the steric hindrance between the lactone carbonyl group and chlorine atom, leading to the inactivity of the compounds [193].

Alizadeh et al. investigated different isodeoxypodophyllotoxin (isoDPT) derivatives. Results showed that the number and position of methoxy groups significantly affected cytotoxicity. While isoDPT exhibited lower activity compared to etoposide, 3′-methoxy, 4′-methoxy and 3′,4′-dimethoxy derivatives showed increased cytotoxicity [197].

Modifications of podophyllotoxin derivatives at the 4′ position showed that replacing the methoxy group with the hydroxy group slightly alters [27] or slightly decreases [188] antimitotic activity. When a glycoside moiety was present at C7, however, even with 4′-OH, the compound was inactive [27]. On the other hand, the hydroxyl group at C4′ is required for inhibitory activity on topoisomerase II and also contributes to a high cytotoxicity [141,188]. All derivatives containing the 4′-hydroxyl group were active in fragmenting DNA—aglycones and glycoside derivatives showed no difference in activity. Additionally, the inhibitory activity of α-peltatin (4′-demethyl derivative of β-peltatin) and inactivity of podophyllotoxin and β-peltatin towards topoisomerase II confirmed the necessity of the 4′-OH group for topoisomerase II inhibition [141]. Moreover, the nature of the substitution at the C4′ position also influences immunomodulatory activity. Compounds with a free phenolic hydroxyl at the 4′-position were more potent compared to those with a methoxy group at the same position, whereas acetylation led to substantially reduced activity [181].

The addition of the aryloxyacetanilide moiety at the 4′-position generally yielded lower cytotoxic activity compared to etoposide, albeit still within the micromolar concentration range. The derivative with a fluorine substituent on the benzene ring emerged as the most potent compound in vitro and exhibited promising activity in vivo [198].

On the other hand, dihydroxy substitution at 3′ and 4′ positions showed no topoisomerase II inhibitory activity, lower cytotoxicity, and lower tubulin polymerization inhibition compared to 4′-OH compounds and DPT [159,188]. Analogues with additional oxygenated rings (dioxole and dioxan) fused to the E ring exhibited lower cytotoxicity compared to podophyllotoxin and were slightly less potent than the ortho-quinone precursor. Moreover, the conversion of the trimethoxyphenyl ring of the acetyl podophyllotoxin series into polyheterocyclic systems significantly reduces cytotoxicity, with the impact becoming more notable as the number of substituents on the phenazine system increases [159]. Substitution other than hydroxyl at C4′ resulted in inactive compounds, except the 4′-benzyl group, with tubulin polymerization inhibitory activity the same as DPT [188]. You et al. showed that esterification of the 4′-OH group increases in vivo antitumor activity [127]. They synthesized several 4′-*O*-alkanoyl and 4′-*O*-carboxyalkanoyl esters of 4′-*O*-demethyl-4-deoxypodophyllotoxin (DDPT). The in vitro cytotoxic activity on A549 and SK-MEL-2 cells demonstrated a dependence on chain length, revealing stronger cytotoxicity with shorter alkyl chains. Conversely, in vivo animal studies yielded generally low antitumor activity for the majority of investigated esters, in comparison to etoposide, except for propanoyl, heptanoyl, 13-carboxyundecanoyl, and 15-carboxypentadecanoyl, which exhibited comparable activity. All investigated esters showed higher antitumor activity than DDPT [127].

## 6. Conclusions

Over the years, various approaches have been devised for clinical applications, leading to the development of several anticancer drugs. However, a significant challenge associated with these agents is their inherent toxicity, resulting from a lack of selectivity. Additionally, the emergence of drug resistance poses a persistent problem. Despite the development of plant-isolated anticancer agents and their derivatives, the quest for a safe, cost-effective, and site-specific anticancer drug remains an ongoing challenge.

This review comprehensively explores the biological activities of *A. sylvestris* lignans, their potential mechanisms of action, and structure–activity relationships. The diverse range of biological activities underscores the pharmacological significance of lignans, warranting further investigation for their potential as novel and selective therapeutic agents. While many of these activities have been demonstrated in vitro, a limited number of studies have included in vivo investigations.

An important aspect not covered in this review is the metabolism of lignans, which can impact their activity through processes such as demethylation, glucuronidation, or hydrolysis in the liver. Understanding the pharmacokinetics and pharmacodynamics of lignans suggests optimal administration routes for their therapeutic use.

Moreover, considerable attention has been focused on investigating alternative sources for lignan production. Considering that podophyllotoxin is synthesized directly from deoxypodophyllotoxin, and given that deoxypodophyllotoxin is a prominent lignan in *A. sylvestris*, this noxious plant emerges as a promising source for podophyllotoxin production.

## Figures and Tables

**Table 1:** Dibenzylbutyrolactones identified in *A. sylvestris*.

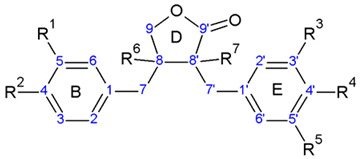								
Name	R^1^	R^2^	R^3^	R^4^	R^5^	R^6^	R^7^	References
(−)-deoxypodorhizone (yatein)	OCH_2_O		OMe	OMe	OMe	αH	βH	[26,29,42,51,53,54,55,65,73,77,79]
matairesinol	OMe	OH	OMe	OH	H	αH	βH	[53,73,77]
thujaplicatin	OMe	OH	OMe	OH	OH	αH	βH	[77]
5-*O*-methylthujaplicatin	OMe	OH	OMe	OH	OMe	αH	βH	[77,80]
4,5-di-*O*-methylthujaplicatin * (thujaplicatin-3,4-dimethylether, hernanol)	OMe	OH	OMe	OMe	OMe	αH	βH	[73,77,80]
dimethylthujaplicatin methyl ether (trimethylthujaplicatin) *	OMe	OMe	OMe	OMe	OMe	αH	βH	[29,73]
pluviatolide *	OCH_2_O		OMe	OH	H	αH	βH	[53,73,77]
bursehernin	OCH_2_O		OMe	OMe	H	αH	βH	[29,52,53,73,77]
arctigenin	OMe	OMe	OMe	OH	H	αH	βH	[29]
(−)-hinokinin	OCH_2_O		OCH_2_O		H	αH	βH	[29,53,65]
dimethylmatairesinol (methylarctigenin)	OMe	OMe	OMe	OMe	H	αH	βH	[29,73]
5-methoxyguayaraol *	OH	OH	OMe	OMe	OMe	αH	βH	[73]

* Tentatively identified compounds are labeled with an asterisk.

**Table 2:** Hydroxy- and oxo-dibenzylbutyrolactones found in *A. sylvestris*.

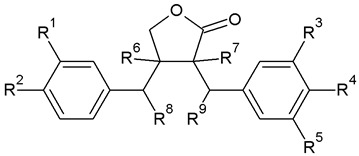										
Name	R^1^	R^2^	R^3^	R^4^	R^5^	R^6^	R^7^	R^8^	R^9^	References
7′-hydroxyyatein	OCH_2_O		OMe	OMe	OMe	αH	βH	OH	H	[29]
8-hydroxy-8-epi-yatein	OCH_2_O		OMe	OMe	OMe	αH	αOH	H	H	[73]
(−)-podorhizol (7-hydroxyyatein)	OCH_2_O		OMe	OMe	OMe	αH	βH	H	βOH	[73]
(+)-podorhizon (7-oxoyatein)	OCH_2_O		OMe	OMe	OMe	αH	βH	H	=O	[73]
8-hydroxy-8′-epi-pluviatolide	OCH_2_O		OMe	OH	H	βH	βOH	H	H	[73]
guayadequiol	OCH_2_O		OMe	OMe	H	βH	βOH	H	H	[73]
epiwikstromol dimethyl ether (8-hydroxy-8-epi-matairesinol)	OMe	OMe	OMe	OMe	H	βH	βOH	H	H	[73]
wikstromol dimethyl ether	OMe	OMe	OMe	OMe	H	βH	αOH	H	H	[73]
8-hydroxy-trimethoxylignano-9,9′-lactone *	(OMe, H)		OMe	OMe	H	H	OH	H	H	[73]
3′,8-dihydroxy-3,4,4′-trimethoxylignano-9,9′-lactone *	OMe	OH	OMe	OMe	H	H	OH	H	H	[73]

* Tentatively identified compounds are labeled with an asterisk.

**Table 3:** Unsaturated dibenzylbutyrolactones found in *A. sylvestris*.

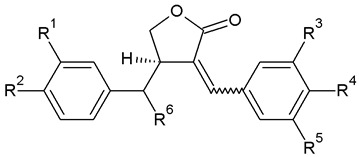							
Name	R^1^	R^2^	R^3^	R^4^	R^5^	R^6^	References
anhydropodorhizol (nemerosin)	OCH_2_O		OMe	OMe	OMe	H	[26,29,42,51,53,54,55,62,73,79]
isochaihulactone	OCH_2_O		OMe	OMe	OMe	H	[73]
isosuchilactone	OMe	OMe	OCH_2_O		H	H	[51]
jatrophan (βH in lactone)	OMe	OMe	OCH_2_O		H	H	[51]
sylvestrin (βH in lactone)	OCH_2_O		OMe	OMe	OMe	H	[42]
kaerophyllin	OCH_2_O		OMe	OMe	H	H	[51,73,79]
isokaerophyllin	OCH_2_O		OMe	OMe	H	H	[73,79]
7′-hydroxyanhydropodorhizol	OCH_2_O		OMe	OMe	OMe	OH	[29]
3,4,5-trimethoxy-3′,4′-dihydroxylign-7-eno-9,9′-lactone *	OH	OH	OMe	OMe	OMe	H	[73,79]
(*E*)-3′-demethyljatrophan	OH	OMe	OCH_2_O		H	H	[73]
(*Z*)-3′-demethyljatrophan	OH	OMe	OCH_2_O		H	H	[73]
(*E*)-7,8-didehydro-dimethylmatairesinol	OMe	OMe	OMe	OMe	H	H	[73]
(*Z*)-7,8-didehydro-dimethylmatairesinol	OMe	OMe	OMe	OMe	H	H	[73]
7,8-didehydroguayarol *	OH	OH	OMe	OMe	H	H	[73]
7,8-didehydroisoarctigenin *	OMe	OH	OMe	OMe	H	H	[73]

* Tentatively identified compounds are labeled with an asterisk.

**Table 4:** Aryltetralins found in *A. sylvestris*.

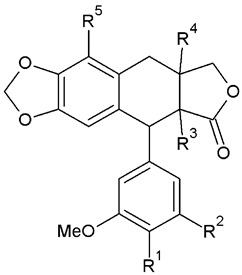						
Name	R^1^	R^2^	R^3^	R^4^	R^5^	References
morelensin	OMe	H	βH	αH	H	[29,52,65,73]
(−)-deoxypodophyllotoxin (anthricin, DPT)	OMe	OMe	βH	αH	H	[26,29,37,42,50,51,52,53,54,55,57,58,65,68,73,79,83,84,85,86,87,88,89,90,91]
isoanthricin	OMe	OMe	βH	αH	H	[50,90]
deoxypicropodophyllotoxin	OMe	OMe	αH	αH	H	[42,90]
α-peltatin	OH	OMe	βH	αH	OH	[54]
β-peltatin	OMe	OMe	βH	αH	OH	[54]
β-peltatin A methyl ether	OMe	OMe	βH	αH	OMe	[54]

**Table 5:** Hydroxy- and oxo- aryltetralins found in *A. sylvestris*.

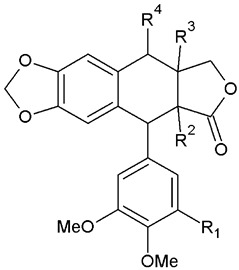					
Name	R^1^	R^2^	R^3^	R^4^	References
podophyllotoxin	OMe	βH	αH	αOH	[29,54,73,79]
picropodophyllotoxin	OMe	αH	αH	αOH	[42,73]
angeloylpodophyllotoxin	OMe	βH	αH	α-C_5_H_7_O_2_	[29,42,52,54,55,73,79]
acetylpodophyllotoxin	OMe	βH	αH	α-CH_3_COO	[73]
5′-demethoxypodophyllotoxin (7-hydroxymorelensin)	H	βH	αH	αOH	[73,79]
podophyllotoxone	OMe	βH	αH	=O	[54,55,73,79]
picropodophyllotoxone (picropodophyllone)	OMe	αH	αH	=O	[73,79]
isopicropodophyllotoxone (isopicropodophyllone)	OMe	βH	βH	=O	[54,55,73]
5′-demethoxypodophyllotoxone	H	βH	αH	=O	[73]
5′-demethoxypicropodophyllotoxone (5′-demethoxypicropodophyllone)	H	αH	αH	=O	[73]
5′-demethoxyisopicropodophyllotoxone (5′-demethoxyisopicropodophyllone)	H	βH	βH	=O	[73]
4-hydroxy-3′,4′,5-trimethoxy-7-oxo-2,7′-cyclolignano-9′,9-lactone *	H	H	H	=O	[73]

* Tentatively identified compounds are labeled with an asterisk.

**Table 6:** Cytotoxic activities of investigated lignans from *A. sylvestris*.

Compound	Cell Line	IC_50_ (µmol/L)	Assay	References
**deoxypodophyllotoxin**	MK-1	0.0025	MTT	[51]
		0.055	MTT	[65]
	HeLa	0.0013	MTT	[51]
		0.083	MTT	[65]
		4.97	CCK-8	[124]
		47.03	MTT	[90]
	B16F10	0.0018	MTT	[51]
		0.21	MTT	[65]
	Colo205	0.24	MTT	[52]
	K562	0.046	MTT	[52]
	A-549	1.38	MTT	[124]
		<0.006	CVS	[125]
		0.0063	CVS	[126]
		0.030	SRB	[127]
		0.053	SRB	[112]
	SiHa	6.01	MTT	[124]
	HL-60	0.47	CCK-8	[124]
	HepG2	30.75	MTT	[90]
	MG-63	36.10	MTT	[90]
	B16	108.67	MTT	[90]
	MCF-7	0.011	MTT	[128]
	MDA-MB-231	0.020	MTT	[128]
		0.0218	MTT	[129]
	SF126	0.014	MTT	[129]
	U-87 MG	0.0151	MTT	[129]
	SGC-7901	0.0197	MTT	[129]
	BGC-823	0.0267	MTT	[129]
	HO-8910	0.0212	MTT	[129]
	SK-0V-3	0.0252	MTT	[129]
	HT-29	0.0187	MTT	[129]
		0.006	CVS	[125]
		0.0063	CVS	[126]
	JeG-3	0.0253	MTT	[129]
	P-388	0.010	CVS	[125]
		0.0063	CVS	[126]
	SK-MEL-2	0.023	SRB	[127]
		0.011	SRB	[112]
**yatein**	MK-1	0.75	MTT	[51]
		1.85	MTT	[65]
	HeLa	2.00	MTT	[51]
		3.15	MTT	[65]
	B16F10	0.87	MTT	[51]
		4.03	MTT	[65]
	A-549	1.1	SRB	[112]
	SK-MEL-2	1.4	SRB	[112]
**nemerosin**	MK-1	1.76	MTT	[51]
	HeLa	1.01	MTT	[51]
	B16F10	1.76	MTT	[51]
**podophyllotoxin**	MK-1	0.014	MTT	[51]
	HeLa	0.006	MTT	[51]
	B16F10	0.0024	MTT	[51]
	A-549	0.012	CVS	[125,130]
	HT-29	0.024	CVS	[125,130]
	P-388	0.012	CVS	[125,130]
**hinokinin**	MK-1	4.72	MTT	[65]
	HeLa	7.29	MTT	[65]
	B16F10	7.68	MTT	[65]
**morelensin**	MK-1	0.24	MTT	[65]
	HeLa	0.14	MTT	[65]
	B16F10	0.23	MTT	[65]
	Colo205	>50	MTT	[52]
	K562	44.70	MTT	[52]
**bursehernin**	Colo205	44.45	MTT	[52]
	K562	1.15	MTT	[52]
**angeloylpodophyllotoxin**	Colo205	0.19	MTT	[52]
	K562	0.037	MTT	[52]
**arctigenin**	AGS	85.75	MTT	[116]
	Hepa 1c1c7	311.02	MTT	[116]
	HL-60	0.180	MTT	[117]
	HepG2	3.49	MTT	[131]
	MH60	1.0	MTT	[118]
**matairesinol**	Hepa 1c1c7	246.65	MTT	[116]
	HL-60	0.114	MTT	[117]
	MH60	8.4	MTT	[118]
**isoanthricin**	HeLa	>377	MTT	[90]
	HepG2	>377	MTT	[90]
	MG-63	87.14	MTT	[90]
	B16	165.13	MTT	[90]
**deoxypicropodophyllotoxin**	HeLa	296.48	MTT	[90]
	A-549	0.13	CVS	[125]
		0.063	CVS	[126]
	HepG2	178.17	MTT	[90]
	HT-29	0.06	CVS	[125]
		0.063	CVS	[126]
	P-388	0.10	CVS	[125]
		0.063	CVS	[126]
**acetylpodophyllotoxin**	A-549	0.625	CVS	[126]
	HT-29	0.625	CVS	[126]
	P-388	0.625	CVS	[126]
**podophyllotoxon**	A-549	1.8	CVS	[126,130]
	HT-29	1.8	CVS	[126,130]
	P-388	1.8	CVS	[126,130]
**picropodophyllotoxon**	A-549	12.0	CVS	[126]
	HT-29	12.0	CVS	[126]
	P-388	12.0	CVS	[126]
**isopicropodophyllotoxone**	A-549	12.1	CVS	[130]
	HT-29	12.1	CVS	[130]
	P-388	6.0	CVS	[130]
	MEL-28	12.1	CVS	[130]
**β-peltatin A methyl ether**	A-549	0.0097	CVS	[126]
	HT-29	0.0097	CVS	[126]
	P-388	0.0097	CVS	[126]
**picropodophyllotoxin**	A-549	6.0	CVS	[126,130]
	HT-29	6.0	CVS	[126,130]
	P-388	6.0	CVS	[126,130]
	MEL-28	6.0	CVS	[130]

## Data Availability

This study generated no new data.

## Abbreviations

*A. sylvestris*	*Anthriscus sylvestris*
A-549	human lung carcinoma cells
AGS	human gastric adenocarcinoma cells
AIF	apoptosis-inducing factor.
Akt	protein kinase B
AMPK	adenosine-monophosphate-activated protein kinase
APT	angeloyl podophyllotoxin
ATG	arctigenin
B16	mouse melanoma cells
B16F10	murine melanoma cell line
BGC-823	human gastric adenocarcinoma cells
BMMC	bone-marrow-derived mast cells
CBS	colchicine-binding site
Cdk	cyclin-dependent kinase
Colo205	colorectal adenocarcinoma cells
COX	cyclooxygenase
DDPT	4′-*O*-demethyl-4-deoxypodophyllotoxin
DPP	deoxypicropodophyllin
DPT	deoxypodophyllotoxin
EGFR	epidermal growth factor receptor
ESCC	esophageal squamous cell carcinoma
ET	etoposide
H460	nonsmall-cell lung cancer cells
HASMC	human aortic smooth muscle cells
HCC827GR	human lung adenocarcinoma cells
HeLa	immortalized cervical cancer cell line
Hepa 1c1c7	mouse hepatoma cells
HepG2	human liver cancer cell line
HIV	human immunodeficiency virus
HL-60	human promyelocytic leukemia cells
HLM	human liver microsomes
HO-8910	human ovarian carcinoma cells
HPV	human papilloma virus
HSV	herpes simplex viruses
HT-29	human colorectal adenocarcinoma cells
HUVECs	human umbilical vein endothelial cells
IFV	influenza A virus
IgE	immunoglobulin E
IGF1R	insulin-like growth factor 1 receptor
IL	interleukin
ILC2s	type 2 innate lymphoid cells
iNOS	inducible nitric oxide synthase
IRF	interferon regulatory factor
JeG-3	human choriocarcinoma cells
K562	human leukemia cells
L5178Y	mouse leukemic cells
LOX	lipoxygenase
LPS	lipopolysaccharide
LT	leukotriene
MAPKs	mitogen-activated protein kinases
MAT	matairesinol
MCF-7	human breast adenocarcinoma cells
MCMV	murine cytomegalovirus
MDA-MB-231	human breast adenocarcinoma cells
MDR	multidrug resistance
MEL-28	human melanoma cells
MET	Mesenchymal–epithelial transition
MG-63	human osteosarcoma cells
MH60	murine hepatoma cells
MK-1	human epithelial cell line
MMP	matrix metalloproteinase
NF-κB	nuclear factor kappa B
NSAID	nonsteroidal anti-inflammatory drugs
NSCLC	nonsmall-cell lung cancer
OA	osteoarthritis
OVA	ovalbumin
P-388	mouse leukemic cells
PANC-1	pancreatic cancer cell line
PARP-1	poly(ADP-ribose) polymerase 1.
PCA	passive cutaneous anaphylaxis
PG	prostaglandin
PI3K	phosphoinositide 3-kinase
PLK	polo-like kinase
PPT	picropodophyllotoxin
PT	podophyllotoxin
RAW264.7 cells	murine macrophage cell line
ROS	reactive oxygen species
SF126	human glioblastoma cell line
SGC-7901	human gastric adenocarcinoma cells
SiHa	human cervical carcinoma cells
SK-MEL-2	human melanoma cells
SK-OV-3	human ovarian adenocarcinoma cells
Th2	T helper cell type 2
TKIs	tyrosine kinase inhibitors
TNF-α	tumor necrosis factor alpha
U-87 MG	human glioblastoma cells
YAT	yatein

## References

[1] Plunkett G.M., Soltis D.E., Soltis P.S. (1996). Evolutionary Patterns in Apiaceae: Inferences Based on MatK Sequence Data. Syst. Bot..

[2] Hultén E., Fries M. (1984). Atlas of North European Vascular Plants North of the Tropic of Cancer.

[3] Walton D.W.H. (1975). European Weeds and Other Alien Species in the Subantarctic. Weed Res..

[4] Olaru O.T., Niţulescu G.M., Orţan A., Dinu-Pîrvu C.E. (2015). Ethnomedicinal, Phytochemical and Pharmacological Profile of *Anthriscus sylvestris* as an Alternative Source for Anticancer Lignans. Molecules.

[5] Townsend C.C. (1984). One New and One Disjunct Variety of Umbelliferae from East Africa. Kew Bull..

[6] Webb C.J., Sykes W.R., Garnock-Jones P.J., Given D.R., Brownsey P.J. (1989). Checklist of Dicotyledons, Gymnosperms, and Pteridophytes Naturalised in New Zealand: Additional Records and Corrections. N. Z. J. Bot..

[7] Hiroe M. (1979). Umbelliferae of World.

[8] Coulter J.M., Rose J.N. (1900). Monograph of the North American Umbelliferae.

[9] Heywood V.H., Heywood V.H. (1971). Systematic Survey of Old World Umbelliferae. The Biology and Chemistry of the Umbelliferae.

[10] Tutin T.G. (1980). Umbellifers of the British Isles. BSBI Handbook No. 2..

[11] Spalik K., Downie S.R. (2001). The Utility of Morphological Characters for Inferring Phylogeny in Scandiceae Subtribe Scandicinae (Apiaceae). Ann. Missouri Bot. Gard..

[12] Tekin M., Civelek S. (2017). A Taxonomic Revision of the Genus *Anthriscus* (Apiaceae) in Turkey. Phytotaxa.

[13] POWO Plants of the World Online. Facilitated by the Royal Botanic Gardens, Kew. http://www.plantsoftheworldonline.org/.

[14] Cannon J.F.M., Tutin T.G., Heywood V.H., Burges N.A., Moore D.M., Valentine D.H., Walters S.M., Webb D.A. (1968). Anthriscus. Flora Europaea: Volume 2 Rosaceae to Umbelliferae.

[15] Hruška K. (1982). Considerazioni Ecologiche, Fitosociologiche e Morfologiche Sul Genere *Anthriscus pers*. G. Bot. Ital..

[16] Hedge I.C., Lamond J.M., Davis P.H., Chamberlain D.F., Matthews V.A. (1972). Umbelliferae. Flora of Turkey and the East Aegean Islands.

[17] Hand R. Euro+Med PlantBase. http://ww2.bgbm.org/EuroPlusMed/.

[18] Muckensturm B., Diyani F., Reduron J.-P. (1995). Grilactone and Other Terpenoids from *Anthriscus nitida*. Biochem. Syst. Ecol..

[19] Deforce K. (2006). The Historical Use of Ladanum. Palynological Evidence from 15th and 16th Century Cesspits in Northern Belgium. Veg. Hist. Archaeobot..

[20] Hadač E. (1978). Ruderal Vegetation of the Broumov Basin, NE. Bohemia. Folia Geobot. Phytotaxon..

[21] Derbyshire S.J., Hoeg R., Haverkort J. (1999). The Biology of Canadian Weeds. 111. *Anthriscus sylvestris* (L.) Hoffm. Can. J. Plant Sci..

[22] Hansson M.L., Persson T.S. (1994). *Anthriscus sylvestris*—A Growing Conservation Problem?. Ann. Bot. Fenn..

[23] Spalik K., Woodel S.R.J. (1994). Regulation of Pollen Production in *Anthriscus sylvestris,* an Andromonoecious Species. Int. J. Plant Sci..

[24] Tamamschian S. (1933). Materials for the Karyosystematics of the Cultivated and Wild Growing Species of the Family Umbelliferae. Tr. Prikl. Bot. Genet. Sel. Ser. 2.

[25] Chatterjee A., Ghosh S., Roy S.C. (1989). A Cytological Survey of Eastern Himalayan Plants III. Cell Chromosom. Res..

[26] Koulman A., Bos R., Medarde M., Pras N., Quax W.J. (2001). A Fast and Simple GC MS Method for Lignan Profiling in *Anthriscus sylvestris* and Biosynthetically Related Plant Species. Planta Med..

[27] Loike J.D., Brewer C.F., Sternlicht H., Gensler W.J., Horwitz S.B. (1978). Structure-Activity Study of the Inhibition of Microtubule Assembly in vitro by Podophyllotoxin and Its Congeners. Cancer Res..

[28] Canel C., Moraes R.M., Dayan F.E., Ferreira D. (2000). Podophyllotoxin. Phytochemistry.

[29] Koulman A., Kubbinga M.E., Batterman S., Woerdenbag H.J., Pras N., Woolley J.G., Quax W.J. (2003). A Phytochemical Study of Lignans in Whole Plants and Cell Suspension Cultures of *Anthriscus sylvestris*. Planta Med..

[30] Rios J.L., Giner R.M., Prieto J.M. (2002). New Findings on the Bioactivity of Lignans. Stud. Nat. Prod. Chem..

[31] Slanina J., Glatz Z. (2004). Separation Procedures Applicable to Lignan Analysis. J. Chromatogr. B.

[32] Ingram D., Sanders K., Kolybaba M., Lopez D. (1997). Case-Control Study of Phyto-Oestrogens and Breast Cancer. Lancet.

[33] Pietinen P., Stumpf K., Männistö S., Kataja V., Uusitupa M., Adlercreutz H. (2001). Serum Enterolactone and Risk of Breast Cancer: A Case-Control Study in Eastern Finland. Cancer Epidemiol. Biomarkers Prev..

[34] Kilkkinen A., Virtamo J., Vartiainen E., Sankila R., Virtanen M.J., Adlercreutz H., Pietinen P. (2004). Serum Enterolactone Concentration Is Not Associated with Breast Cancer Risk in a Nested Case-Control Study. Int. J. Cancer.

[35] Vanharanta M., Voutilainen S., Rissanen T.H., Adlercreutz H., Salonen J.T. (2003). Risk of Cardiovascular Disease-Related and All-Cause Death According to Serum Concentrations of Enterolactone: Kuopio Ischaemic Heart Disease Risk Factor Study. Arch. Intern. Med..

[36] Podwyssotzki V. (1880). Pharmakologische Studien Über Podophyllum Peltatum. Arch. Exp. Pathol. Pharmakol..

[37] Noguchi T., Kawanami M. (1940). Studies on the Constituents of *Anthriscus sylvestris* Hoffm. Yakugaku Zasshi.

[38] Sackett D.L. (1993). Podophyllotoxin, Steganacin and Combretastatin: Natural Products That Bind at the Colchicine Site of Tubulin. Pharmacol. Ther..

[39] Hartwell J.L., Schrecker A.W. (1958). The Chemistry of Podophyllum. Fortschritte Chem. Org. Naturstoffe.

[40] Kozawa M., Baba K., Matsuyama Y., Kido T., Sakai M., Takemoto T. (1982). Components of the Root of *Anthriscus sylvestris* Hoffm. II. Insecticidal Activity. Chem. Pharm. Bull..

[41] Lee S.H., Son M.J., Ju H.K., Lin C.X., Moon T.C., Choi H., Son J.K., Chang H.W. (2004). Dual Inhibition of Cyclooxygenases-2 and 5-Lipoxygenase by Deoxypodophyllotoxin in Mouse Bone Marrow-Derived Mast Cells. Biol. Pharm. Bull..

[42] Jeong G., Kwon O., Park B., Oh S., Ahn K., Chang M., Oh W.K., Kim J., Min B., Kim Y. (2007). Lignans and Coumarins from the Roots of *Anthriscus sylvestris* and Their Increase of Caspase-3 Activity in HL-60 Cells. Biol. Pharm. Bull..

[43] Milovanovic M., Banjac N., Vucelic-Radovic B. (2009). Functional Food: Rare Herbs, Seeds and Vegetable Oils as Sources of Flavors and Phytosterols. J. Agric. Sci. Belgrade.

[44] Allen D.E., Hatfield G. (2004). Medicinal Plants in Folk Tradition: An Ethnobotany of Britain and Ireland.

[45] Wahida B., Amor M., Nabil C., Rai M., Charya D., Luis Rios J. (2011). An Inventory of Ethnomedicinal Plants Used in Tunisia. Ethnomedicinal Plants: Revitalization of Traditional Knowledge of Herbs.

[46] Gross G. (2001). Clinical Aspects and Therapy of Anogenital Warts and Papillomavirus-Associated Lesions. Hautarzt.

[47] Carlström K., Hedin P., Jönsson L., Lerndal T., Lien J., Weitoft T., Axelson M. (2000). Endocrine Effects of the Podophyllotoxine Derivative Drug CPH 82 (Reumacon®) in Patients with Rheumatoid Arthritis. Scand. J. Rheumatol..

[48] Özqeltk H. (1994). Notes on Economic Plants. Econ. Bot..

[49] Orčić D., Berežni S., Mimica-Dukić N. (2022). Quantitative HPLC-UV Study of Lignans in *Anthriscus sylvestris*. Molecules.

[50] Kozawa M., Morita N., Hata K. (1978). Chemical Components of the Roots of *Anthriscus sylvestris* Hoffm. I. Structures of an Acyloxycarboxylic Acid and a New Phenylpropanoidester, Anthriscusin. Yakugaku Zasshi.

[51] Ikeda R., Nagao T., Okabe H., Nakano Y., Matsunaga H., Katano M., Mori M. (1998). Antiproliferative Constituents in Umbelliferae Plants. III. Constituents in Teh Root and the Ground Part of *Anthriscus sylvestris* Hoffm. Chem. Pharm. Bull..

[52] Lim Y., Leem M., Shin D., Chang H., Hong S., Moon E., Lee D., Yoon S., Woo W. (1999). Cytotoxic Constituents from the Roots of *Anthriscus sylvestris*. Arch. Pharm. Res..

[53] Suzuki S., Sakakibara N., Umezawa T., Shimada M. (2002). Survey and Enzymatic Formation of Lignans of *Anthriscus sylvestris*. J. Wood Sci..

[54] Hendrawati O., Woerdenbag H.J., Michiels P.J.A., Aantjes H.G., van Dam A., Kayser O. (2011). Identification of Lignans and Related Compounds in *Anthriscus sylvestris* by LC-ESI-MS/MS and LC-SPE-NMR. Phytochemistry.

[55] Seegers C.L.C., Tepper P.G., Setroikromo R., Quax W.J. (2018). Cytotoxic Deoxypodophyllotoxin Can Be Extracted in High Purity from *Anthriscus sylvestris* Roots by Supercritical Carbon Dioxide. Planta Med..

[56] Kozawa M., Morita N., Hata K. (1978). Structure of Anthriscusin, a New Phenylpropanoid Ester from the Roots of *Anthriscus sylvestris* Hoffm. Chem. Pharm. Bull..

[57] Kurihara T., Kikuchi M., Suzuki S., Hisamichi S. (1978). Studies on the Constituents of *Anthriscus sylvestris* Hoffm. I. On the Components of the Radix. Yakugaku Zasshi.

[58] Du C., Lei B., Ning N., Fan J., Zhang X., Ma C., Jiang H. (2020). A New Phenylpropanoid Ester from the Roots of *Anthriscus sylvestris* and Its Chemotaxonomic Significance. Biochem. Syst. Ecol..

[59] Liu Y., Cao Y., Niu Y., Zheng Y., Chen X., Ren Y., Fan X., Li X., Ma X., Zheng X. (2023). Diarylpentanoids and Phenylpropanoids from the Roots of *Anthriscus sylvestris* (L.) Hoffm. Phytochemistry.

[60] Bos R., Koulman A., Woerdenbag H.J., Quax W.J., Pras N. (2002). Volatile Components from *Anthriscus sylvestris* (L.) Hoffm. J. Chromatogr. A.

[61] Kramer M., Mühleis A., Conrad J., Leitenberger M., Beifuss U., Carle R., Kammerer D.R. (2011). Quantification of Polyacetylenes in Apiaceous Plants by High-Performance Liquid Chromatography Coupled with Diode Array Detection. Z. Naturforsch.

[62] Dall’Acqua S., Giorgetti M., Cervellati R.I., Nnocenti G. (2006). Deoxypodophyllotoxin Content and Antioxidant Activity of Aerial Parts of *Anthriscus sylvestris* Hoffm. Z. Naturforsch.

[63] Milovanovic M., Stefanovic M., Djermanovic V., Milovanovic J. (1996). Some Chemical Constituents of *Anthriscus sylvestris*. J. Herbs. Spices Med. Plants.

[64] Milovanovic M., Picuric-Jovanovic K., Vucelic-Radovic B., Vrbaski Z. (1996). Antioxidant Effects of Flavonoids of *Anthriscus sylvestris* in Lard. J. Am. Oil Chem. Soc..

[65] Ikeda R., Nagao T., Okabe H., Nakano Y., Matsunaga H., Katano M., Mori M. (1998). Antiproliferative Constituents in Umbelliferae Plants. IV. Constituents in the Fruits of *Anthriscus sylvestris* Hoffm. Chem. Pharm. Bull..

[66] Janković M., Berežni S., Orčić D. (2023). Quantitative Analysis of Lignans from the Fruits of Wild Chervil (*Anthriscus sylvestris* (L.)Hoffm.). FACTA Univ. Ser. Physics, Chem. Technol..

[67] Janković M., Berežni S., Orčić D. (2023). Lignan Profile in Fruits of Wild Chervil (*Anthriscus sylvestris* (L.) Hoffm.). FACTA Univ. Ser. Physics, Chem. Technol..

[68] Kurihara T., Kikuchi M. (1979). Studies on the Constituents of *Anthriscus sylvestris* Hoffm. II. On the Components of the Flowers and Leaves. Yakugaku Zasshi.

[69] Borg-Karlson A., Valterová I., Anders Nilsson L. (1994). Volatile Compounds from Flowers of Six Species in the Family Apiaceae: Bouquets for Different Pollinators?. Phytochemistry.

[70] Sólyomváry A., Béni S., Boldizsár I. (2017). Dibenzylbutyrolactone Lignans—A Review of Their Structural Diversity, Biosynthesis, Occurrence, Identification and Importance. Mini-Rev. Med. Chem..

[71] Guerram M., Jiang Z.Z., Zhang L.Y. (2012). Podophyllotoxin, a Medicinal Agent of Plant Origin: Past, Present and Future. Chin. J. Nat. Med..

[72] Umezawa T. (2003). Diversity in Lignan Biosynthesis. Phytochem. Rev..

[73] Orčić D., Berežni S., Škorić D., Mimica-Dukić N. (2021). Comprehensive Study of *Anthriscus sylvestris* Lignans. Phytochemistry.

[74] Ayres D.C., Loike J.D. (1990). Lignans Chemical, Biological and Clinical Properties.

[75] Dewick P.M., Jackson D.E. (1981). Cytotoxic Lignans from Podophyllum, and the Nomenclature of Aryltetralin Lignans. Phytochemistry.

[76] Cui Q., Du R., Liu M., Rong L. (2020). Lignans and Their Derivatives from Plants as Antivirals. Molecules.

[77] Sakakibara N., Suzuki S., Umezawa T., Shimada M. (2003). Biosynthesis of Yatein in *Anthriscus sylvestris*. Org. Biomol. Chem..

[78] Koulman A. (2003). Podophyllotoxin: A Study of the Biosynthesis, Evolution, Function and Use of Podophyllotoxin and Related Lignans.

[79] Orčić D. (2010). Vrste Tribusa Scandiceae (Apiaceae Lindley 1836, Subfam. Apioideae) Potencijalni Izvor Biološki i Farmakološki Aktivnih Sekundarnih Biomolekula.

[80] Ragamustari S.K., Nakatsubo T., Hattori T., Ono E., Kitamura Y., Suzuki S., Yamamura M., Umezawa T. (2013). A Novel O-Methyltransferase Involved in the First Methylation Step of Yatein Biosynthesis from Matairesinol in *Anthriscus sylvestris*. Plant Biotechnol..

[81] Kamil W.M., Dewick P.M. (1986). Biosynthetic Relationship of Aryltetralin Lactone Lignans to Dibenzylbutyrolactone Lignans. Phytochemistry.

[82] Jackson D.E., Dewick P.M. (1984). Biosynthesis of Podophyllum Lignans—II. Interconversions of Aryltetralin Lignans in Podophyllum Hexandrum. Phytochemistry.

[83] Lin C.X., Son M.J., Ju H.K., Moon T.C., Lee E., Kim S.H., Kim M., Son J.K., Lee S.H., Chang H.W. (2004). Deoxypodophyllotoxin, a Naturally Occurring Lignan, Inhibits the Passive Cutaneous Anaphylaxis Reaction. Planta Med..

[84] Lin C.X., Lee E., Jin M.H., Yook J., Quan Z., Ha K., Moon T.C., Kim M.J., Kim K.J., Lee S.H. (2006). Deoxypodophyllotoxin (DPT) Inhibits Eosinophil Recruitment into the Airway and Th2 Cytokine Expression in an OVA-Induced Lung Inflammation. Planta Med..

[85] Jin M., Moon T.C., Quan Z., Lee E., Kim Y.K., Yang J.H., Suh S.J., Jeong T.C., Lee S.H., Kim C.H. (2008). The Naturally Occurring Flavolignan, Deoxypodophyllotoxin, Inhibits Lipopolysaccharide-Induced INOS Expression through the NF-κB Activation in RAW264.7 Macrophage Cells. Biol. Pharm. Bull..

[86] Yong Y., Shin S.Y., Lee Y.H., Lim Y. (2009). Antitumor Activity of Deoxypodophyllotoxin Isolated from *Anthriscus sylvestris*: Induction of G2/M Cell Cycle Arrest and Caspase-Dependent Apoptosis. Bioorg. Med. Chem. Lett..

[87] Quan G.H., Chin Y.W., Lee H.K., Oh S.R. (2010). Preparative Isolation and Purification of Deoxypodophyllotoxin from the Rhizomes of *Anthriscus sylvestris* by High-Speed Counter-Current Chromatography. J. Korean Soc. Appl. Biol. Chem..

[88] Jung C.H., Kim H., Ahn J., Jung S.K., Um M.Y., Son K.H., Kim T.W., Ha T.Y. (2013). Anthricin Isolated from *Anthriscus sylvestris* (L.) Hoffm. Inhibits the Growth of Breast Cancer Cells by Inhibiting Akt/mTOR Signaling, and Its Apoptotic Effects Are Enhanced by Autophagy Inhibition. Evid.-Based Complement. Altern. Med..

[89] Cho E.J., Choi J.M., Kim H.M., Choi K., Ku J., Park K.W., Kim J., Lee S. (2013). Antibacterial Activity and Protective Effect against Gastric Cancer by *Anthriscus sylvestris* Fractions. Hortic. Environ. Biotechnol..

[90] Chen H., Jiang H.Z., Li Y.C., Wei G.Q., Geng Y., Ma C.Y. (2014). Antitumor Constituents from *Anthriscus sylvestris* (L.) Hoffm. Asian Pacific J. Cancer Prev..

[91] Kim H.S., Lee A.Y., Moon B.C., Kim W.J., Choi G. (2019). Ultrasonic-Assisted Extraction Process and Method Validation for Deoxypodophyllotoxin from the Roots of *Anthriscus sylvestris*: Application of Response Surface Methodology and UPLC–PDA–QDa. Acta Chromatogr..

[92] Velescu B.Ş., Anuţa V., Nițulescu G.M., Olaru O.T., Orțan A., Ionescu D., Ghica M.V., Drăgoi C.M., Pîrvu C.E.D. (2017). Pharmaceutical Assesment of Romanian Crops of *Anthriscus sylvestris* (Apiaceae). Farmacia.

[93] Lee S.A., Moon S., Han S.H., Hwang E.J., Park B., Kim J., Kim D.K., Kim C.S. (2018). Chondroprotective Effects of Aqueous Extract of *Anthriscus sylvestris* Leaves on Osteoarthritis in vitro and in vivo through MAPKs and NF-κB Signaling Inhibition. Biomed. Pharmacother..

[94] Lee S.A., Moon S.M., Han S.H., Hwang E.J., Hong J.H., Park B.R., Choi M.S., Ahn H., Kim J.S., Kim H.J. (2018). In vivo and in vitro Anti-Inflammatory Effects of Aqueous Extract of *Anthriscus sylvestris* Leaves. J. Med. Food.

[95] Vane J.R. (1971). Inhibition of Prostaglandin Synthesis as a Mechanism of Action for Aspirin-like Drugs. Nat. New Biol..

[96] Whittle B.J.R. (1981). Arachidonic Acid Metabolites and the Gastro-Intestinal Toxicity of Anti-Inflammatory Agents. Prostaglandins.

[97] Schneider I., Bucar F. (2005). Lipoxygenase Inhibitors from Natural Plant Sources. Part 2: Medicinal Plants with Inhibitory Activity on Arachidonate 12-Lipoxygenase, 15-Lipoxygenase and Leukotriene Receptor Antagonists. Phyther. Res..

[98] León B., Ballesteros-Tato A. (2021). Modulating Th2 Cell Immunity for the Treatment of Asthma. Front. Immunol..

[99] Drazen J.M., Arm J.P., Austen K.F. (1996). Sorting out the Cytokines of Asthma. J. Exp. Med..

[100] Bousquet J., Chanez P., Lacoste J.Y., Barneon G., Ghavanian N., Enander I., Venge P., Ahlstedt S., Simony-Lafontaine J., Godard P. (1990). Eosinophilic Inflammation in Asthma. N. Engl. J. Med..

[101] Maarsingh H., Dekkers B.G.J., Zuidhof A.B., Bos I.S.T., Menzen M.H., Klein T., Flik G., Zaagsma J., Meurs H. (2011). Increased Arginase Activity Contributes to Airway Remodelling in Chronic Allergic Asthma. Eur. Respir. J..

[102] Hamad A.M., Knox A.J. (2001). Mechanisms Mediating the Antiproliferative Effects of Nitric Oxide in Cultured Human Airway Smooth Muscle Cells. FEBS Lett..

[103] Sharma J.N., Al-Omran A., Parvathy S.S. (2007). Role of Nitric Oxide in Inflammatory Diseases. Inflammopharmacology.

[104] Kim S.B., Lee A.Y., Chun J.M., Lee A.R., Kim H.S., Seo Y.S., Moon B.C., Kwon B.I. (2019). *Anthriscus sylvestris* Root Extract Reduces Allergic Lung Inflammation by Regulating Interferon Regulatory Factor 4-Mediated Th2 Cell Activation. J. Ethnopharmacol..

[105] An M., Oh M., Park K.T., Seon K.H., Jo J.E., Lee S.K., Kim J.K., Shin K.S., Koh J.H., Lim Y.H. (2021). Anti-Asthma and Antitussive Effects of a Fermented Extract of a Mixture of Ramulus Mori, *Anthriscus sylvestris*, and *Salvia plebeian*. Food Sci. Biotechnol..

[106] Suh S.J., Kim J.R., Jin U.H., Choi H.S., Chang Y.C., Lee Y.C., Kim S.H., Lee I.S., Moon T.C., Chang H.W. (2009). Deoxypodophyllotoxin, Flavolignan, from *Anthriscus sylvestris* Hoffm. Inhibits Migration and MMP-9 via MAPK Pathways in TNF-α-Induced HASMC. Vascul. Pharmacol..

[107] Farina A.R., Mackay A.R. (2014). Gelatinase B/MMP-9 in Tumour Pathogenesis and Progression. Cancers.

[108] Wang Y., Wang B., Guerram M., Sun L., Shi W., Tian C., Zhu X., Jiang Z., Zhang L. (2015). Deoxypodophyllotoxin Suppresses Tumor Vasculature in HUVECs by Promoting Cytoskeleton Remodeling through LKB1-AMPK Dependent Rho A Activation. Oncotarget.

[109] Choi H., Lee J., Shin H., Lee B., Chang I., Hwang J. (2004). Deoxypodophyllotoxin Reduces Skin Pigmentation of Brown Guinea Pigs. Planta Med..

[110] Briganti S., Camera E., Picardo M. (2003). Chemical and Instrumental Approaches to Treat Hyperpigmentation. Pigment Cell Res..

[111] Muto N., Tomokuni T., Haramoto M., Tatemoto H., Nakanishi T., Inatomi Y., Murata H., Inada A. (2008). Isolation of Apoptosis- and Differentiation-Inducing Substances toward Human Promyelocytic Leukemia HL-60 Cells from Leaves of *Juniperus taxifolia*. Biosci. Biotechnol. Biochem..

[112] Kim Y., You Y.J., Nam N.H., Ahn B.Z. (2002). 2,3-Dibenzylbutyrolactones and 1,2,3,4-Tetrahydro-2-Naphthoic Acid y-Lactones: Structure and Activity Relationship in Cytotoxic Activity. Arch. Pharm. Res..

[113] Srivastava V., Singh Negi A.S., Kumar J.K., Gupta M.M., Khanuja S.P.S. (2005). Plant-Based Anticancer Molecules: A Chemical and Biological Profile of Some Important Leads. Bioorg. Med. Chem..

[114] Inamori Y., Kubo M., Tsujibo H., Ogawa M., Baba K., Kozawa M., Fujita E. (1986). The Biological Activities of Podophyllotoxin Compounds. Chem. Pharm. Bull..

[115] Levy R.K., Hall I.H., Lee K.H. (1983). Antitumor Agents LXII: Synthesis and Biological Evaluation of Podophyllotoxin Esters and Related Derivatives. J. Pharm. Sci..

[116] Kang K., Lee H.J., Kim C.Y., Lee S.B., Tunsag J., Batsuren D., Nho C.W. (2007). The Chemopreventive Effects of *Saussurea salicifolia* through Induction of Apoptosis and Phase II Detoxification Enzyme. Biol. Pharm. Bull..

[117] Hirano T., Gotoh M., Oka K. (1994). Natural Flavonoids and Lignans Are Potent Cytostatic Agents against Human Leukemic HL-60 Cells. Life Sci..

[118] Matsumoto T., Hosono-Nishiyama K., Yamada H. (2006). Antiproliferative and Apoptotic Effects of Butyrolactone Lignans from Arctium Lappa on Leukemic Cells. Planta Med..

[119] Awale S., Lu J., Kalauni S.K., Kurashima Y., Tezuka Y., Kadota S., Esumi H. (2006). Identification of Arctigenin as an Antitumor Agent Having the Ability to Eliminate the Tolerance of Cancer Cells to Nutrient Starvation. Cancer Res..

[120] Takasaki M., Konoshima T., Komatsu K., Tokuda H., Nishino H. (2000). Anti-Tumor-Promoting Activity of Lignans from the Aerial Part of *Saussurea medusa*. Cancer Lett..

[121] Cho J.Y., Kim A.R., Yoo E.S., Baik K.U., Park M.H. (1999). Immunomodulatory Effect of Arctigenin, a Lignan Compound, on Tumour Necrosis Factor-α and Nitric Oxide Production, and Lymphocyte Proliferation. J. Pharm. Pharmacol..

[122] Hausott B., Greger H., Marian B. (2003). Naturally Occurring Lignans Efficiently Induce Apoptosis in Colorectal Tumor Cells. J. Cancer Res. Clin. Oncol..

[123] Ma D., Lu B., Feng C., Wang C., Wang Y., Luo T., Feng J., Jia H., Chi G., Luo Y. (2016). Deoxypodophyllotoxin Triggers Parthanatos in Glioma Cells via Induction of Excessive ROS. Cancer Lett..

[124] Chen S., Gao Y., Zhou N., Liu J., Huang W., Hui L., Jin Y., Jin Y. (2011). Carbamates of 4′-Demethyl-4-Deoxypodophyllotoxin- Synthesis, Cytotoxicity and Cell Cycle Effects. Bioorg. Med. Chem. Lett..

[125] Gordaliza M., Castro M.A., García-Grávalos M.D., Ruiz P., Del Corral J.M.M., San Feliciano A. (1994). Antineoplastic and Antiviral Activities of Podophyllotoxin Related Lignans. Arch. Pharm..

[126] San Feliciano A., Gordaliza M., Miguel Del Corral J.M., Castro M.A., Garcia-Gravalos M.D., Ruiz-Lazaro P. (1993). Antineoplastic and Antiviral Activities of Some Cyclolignans. Planta Med..

[127] You Y., Kim Y., Nam N., Bang S., Ahn B. (2004). Alkyl and Carboxylalkyl Esters of 4′-Demethyl-4-Deoxypodophyllotoxin: Synthesis, Cytotoxic, and Antitumor Activity. Eur. J. Med. Chem..

[128] Khaled M., Belaaloui G., Jiang Z.Z., Zhu X., Zhang L.Y. (2017). Deoxypodophyllotoxin, a Semi-Synthetic Compound from *Dysosma versipellis*, Induces Selective Cell Death in Human Breast Cancer Cell Lines. Med. Chem. Res..

[129] Guerram M., Jiang Z.Z., Sun L., Zhu X., Zhang L.Y. (2015). Antineoplastic Effects of Deoxypodophyllotoxin, a Potent Cytotoxic Agent of Plant Origin, on Glioblastoma U-87 MG and SF126 Cells. Pharmacol. Rep..

[130] Gordaliza M., Miguel Del Corral J.M., Angeles Castro M., García-García P.A., San Feliciano A. (2001). Cytotoxic Cyclolignans Related to Podophyllotoxin. Il Farm..

[131] Moritani S., Nomura M., Takeda Y., Miyamoto K. (1996). Cytotoxic Components of Bardanae Fructus (Goboshi). Biol. Pharm. Bull..

[132] McLoughlin E.C., O’Boyle N.M. (2020). Colchicine-Binding Site Inhibitors from Chemistry to Clinic: A Review. Pharmaceuticals.

[133] Dominguez-Brauer C., Thu K.L., Mason J.M., Blaser H., Bray M.R., Mak T.W. (2015). Targeting Mitosis in Cancer: Emerging Strategies. Mol. Cell.

[134] Zang X., Wang G., Cai Q., Zheng X., Zhang J., Chen Q., Wu B., Zhu X., Hao H., Zhou F. (2018). A Promising Microtubule Inhibitor Deoxypodophyllotoxin Exhibits Better Efficacy to Multidrug-Resistant Breast Cancer than Paclitaxel via Avoiding Efflux Transport. Drug Metab. Dispos..

[135] Shin S.Y., Yong Y., Lee Y.H. (2010). Effect of Deoxypodophyllotoxin Isolated from *Anthriscus sylvestris* Roots on the Expression of Cell Cycle-Regulatory Proteins in HeLa Cells. J. Appl. Biol. Chem..

[136] Malumbres M., Barbacid M. (2005). Mammalian Cyclin-Dependent Kinases. Trends Biochem. Sci..

[137] Khaled M., Jiang Z.Z., Zhang L.Y. (2013). Deoxypodophyllotoxin: A Promising Therapeutic Agent from Herbal Medicine. J. Ethnopharmacol..

[138] Wang Y.R., Xu Y., Jiang Z.Z., Guerram M., Wang B., Zhu X., Zhang L.Y. (2015). Deoxypodophyllotoxin Induces G2/M Cell Cycle Arrest and Apoptosis in SGC-7901 Cells and Inhibits Tumor Growth in vivo. Molecules.

[139] Wu M., Jiang Z., Duan H., Sun L., Zhang S., Chen M., Wang Y., Gao Q., Song Y., Zhu X. (2013). Deoxypodophyllotoxin Triggers Necroptosis in Human Non-Small Cell Lung Cancer NCI-H460 Cells. Biomed. Pharmacother..

[140] MacRae W.D., Towers G.H.N. (1984). Biological of Lignans. Phytochemistry.

[141] Loike J.D., Horwitz S.B. (1976). Effect of VP-16-213 on the Intracellular Degradation of DNA in HeLa Cells. Biochemistry.

[142] Grieder A., Maurer R., Stähelin H. (1974). Effect of an Epipodophyllotoxin Derivative (VP 16-213) on Macromolecular Synthesis and Mitosis in Mastocytoma Cells in vitro. Cancer Res..

[143] Huang C.C., Hou Y., Wang J.J. (1973). Effects of a New Antitumor Agent, Epipodophyllotoxin, on Growth and Chromosomes in Human Hematopoietic Cell Lines. Cancer Res..

[144] Lakhani S.A., Masud A., Kuida K., Porter G.A., Booth C.J., Mehal W.Z., Inayat I., Flavell R.A. (2006). Caspases 3 and 7: Key Mediators of Mitochondrial Events of Apoptosis. Science.

[145] Ponder K.G., Boise L.H. (2019). The Prodomain of Caspase-3 Regulates Its Own Removal and Caspase Activation. Cell Death Discov..

[146] Nitulescu G.M., Margina D., Juzenas P., Peng Q., Olaru O.T., Saloustros E., Fenga C., Spandidos D.A., Libra M., Tsatsakis A.M. (2016). Akt Inhibitors in Cancer Treatment: The Long Journey from Drug Discovery to Clinical Use (Review). Int. J. Oncol..

[147] Luo H.R., Hattori H., Hossain M.A., Hester L., Huang Y., Lee-Kwon W., Donowitz M., Nagata E., Snyder S.H. (2003). Akt as a Mediator of Cell Death. Proc. Natl. Acad. Sci. USA.

[148] Grunt T.W., Mariani G.L. (2013). Novel Approaches for Molecular Targeted Therapy of Breast Cancer: Interfering with PI3K/AKT/MTOR Signaling. Curr. Cancer Drug Targets.

[149] Park B., Lee S.A., Moon S.M., Kim C.S. (2018). Anthricin-induced Caspase-dependent Apoptosis through IGF1R-PI3K-AKT Pathway Inhibition in A549 Human Non-small Lung Cancer Cells. Oncol. Rep..

[150] Baserga R., Peruzzi F., Reiss K. (2003). The IGF-1 Receptor in Cancer Biology. Int. J. Cancer.

[151] Kwak A.W., Lee M.H., Yoon G., Cho S.S., Choi J.S., Chae J.I., Shim J.H. (2020). Deoxypodophyllotoxin, a Lignan from *Anthriscus sylvestris*, Induces Apoptosis and Cell Cycle Arrest by Inhibiting the Egfr Signaling Pathways in Esophageal Squamous Cell Carcinoma Cells. Int. J. Mol. Sci..

[152] Johnson M., Chiara M., Mok T., Mitsudomi T. (2022). Treatment Strategies and Outcomes for Patients with EGFR-Mutant Non-Small Cell Lung Cancer Resistant to EGFR Tyrosine Kinase Inhibitors: Focus on Novel Therapies. Lung Cancer.

[153] Kim H.S., Oh H.N., Kwak A.W., Kim E., Lee M.H., Seo J.H., Cho S.S., Yoon G., Chae J.I., Shim J.H. (2021). Deoxypodophyllotoxin Inhibits Cell Growth and Induces Apoptosis in Gefitinib-Resistant Non-Small Lung Cancer Cells by Dual-Targeting EGFR and MET. J. Microbiol. Biotechnol..

[154] Lee J.Y., Kang B.Y., Jung S.J., Kwak A.W., Lee S.O., Park J.W. (2022). Picropodophyllotoxin Inhibits Cell Growth and Induces Apoptosis in Gefitinib-Resistant Non-Small Lung Cancer Cells by Dual-Targeting EGFR and MET. Biomol. Ther..

[155] Negut I., Grumezescu V., Grumezescu A.M., Bîrcă A.C., Holban A.M., Urzica I., Avramescu S.M., Gălăţeanu B., Hudiţă A. (2020). Nanostructured Thin Coatings Containing *Anthriscus sylvestris* Extract with Dual Bioactivity. Molecules.

[156] Saitoh T., Kuramochi K., Imai T., Takata K., Takehara M., Kobayashi S., Sakaguchi K., Sugawara F. (2008). Podophyllotoxin Directly Binds a Hinge Domain in E2 of HPV and Inhibits an E2/E7 Interaction in vitro. Bioorg. Med. Chem..

[157] Nishimura A., Ono T., Ishimoto A., Dowhanick J.J., Frizzell M.A., Howley P.M., Sakai H. (2000). Mechanisms of Human Papillomavirus E2-Mediated Repression of Viral Oncogene Expression and Cervical Cancer Cell Growth Inhibition. J. Virol..

[158] Bedows E., Hatfield G.M. (1982). An Investigation of the Antiviral Activity of *Podophyllum peltatum*. J. Nat. Prod..

[159] Castro M.A., Miguel Del Corral J.M., Gordaliza M., Gómez-Zurita M.A., De La Puente M.L., Betancur-Galvis L.A., Sierra J., San Feliciano A. (2003). Synthesis, Cytotoxicity and Antiviral Activity of Podophyllotoxin Analogues Modified in the E-Ring. Eur. J. Med. Chem..

[160] Hammonds T.R., Denyer S.P., Jackson D.E., Irving W.L. (1996). Studies to Show That with Podophyllotoxin the Early Replicative Stages of Herpes Simplex Virus Type 1 Depend upon Functional Cytoplasmic Microtubules. J. Med. Microbiol..

[161] MacRae W.D., Hudson J.B.N., Towers G.H. (1989). The Antiviral Action of Lignans. Planta Med..

[162] Charlton J.L. (1998). Antiviral Activity of Lignans. J. Nat. Prod..

[163] Hayashi K., Narutaki K., Nagaoka Y., Hayashi T., Uesato S. (2010). Therapeutic Effect of Arctiin and Arctigenin in Immunocompetent and Immunocompromised Mice Infected with Influenza A Virus. Biol. Pharm. Bull..

[164] Eich E., Pertz H., Kaloga M., Schulz J., Fesen M.R., Mazumder A., Pommier Y. (1996). (−)-Arctigenin as a Lead Structure for Inhibitors of Human Immunodeficiency Virus Type-1 Integrase. J. Med. Chem..

[165] Schröder H.C., Merz H., Steffen R., Müller W.E.G., Sarin P.S., Trumm S., Schulz J., Eich E. (1990). Differential in vitro Anti-HIV Activity of Natural Lignans. Z. Naturforsch.—Sect. C J. Biosci..

[166] Eich E., Schulz J., Trumm S., Sarin P.S., Maidof A., Merz H., Schröder H.C., Muller W.E.G. (1990). Lignanolides: Novel In vitro Anti-HIV Active. Planta Med..

[167] Kim Y., Hollenbaugh J.A., Kim D.H., Kim B. (2011). Novel PI3K/Akt Inhibitors Screened by the Cytoprotective Function of Human Immunodeficiency Virus Type 1 Tat. PLoS ONE.

[168] Liu Y., Wang Y., Yamakuchi M., Masuda S., Tokioka T., Yamaoka S., Maruyama I., Kitajima I. (2001). Phosphoinositide-3 Kinase-PKB/Akt Pathway Activation Is Involved in Fibroblast Rat-1 Transformation by Human T-Cell Leukemia Virus Type I Tax. Oncogene.

[169] Esteves F., Rueff J., Kranendonk M. (2021). The Central Role of Cytochrome P450 in Xenobiotic Metabolism—A Brief Review on a Fascinating Enzyme Family. J. Xenobiotics.

[170] Julsing M.K., Vasilev N.P., Schneidman-Duhovny D., Muntendam R., Woerdenbag H.J., Quax W.J., Wolfson H.J., Ionkova I., Kayser O. (2008). Metabolic Stereoselectivity of Cytochrome P450 3A4 towards Deoxypodophyllotoxin: In Silico Predictions and Experimental Validation. Eur. J. Med. Chem..

[171] Franklin M.R. (1971). The Enzymic Formation of a Methylenedioxyphenyl Derivative Exhibiting an Isocyanide-like Spectrum with Reduced Cytochrome P-450 in Hepatic Microsomes. Xenobiotica.

[172] Lee S.K., Kim Y., Jin C., Lee S.H., Kang M.J., Jeong T.C., Jeong S.Y., Kim D.H., Yoo H.H. (2010). Inhibitory Effects of Deoxypodophyllotoxin from *Anthriscus sylvestris* on Human CYP2C9 and CYP3A4. Planta Med..

[173] Xu P., Sun Q., Wang X., Zhang S., An S., Cheng J., Gao R., Xiao H. (2010). Pharmacological Effect of Deoxypodophyllotoxin: A Medicinal Agent of Plant Origin, on Mammalian Neurons. Neurotoxicology.

[174] Djamgoz M.B.A., Fraser S.P., Brackenbury W.J. (2019). In vivo Evidence for Voltage-Gated Sodium Channel Expression in Carcinomas and Potentiation of Metastasis. Cancers.

[175] Lopez-Charcas O., Pukkanasut P., Velu S.E., Brackenbury W.J., Hales T.G., Besson P., Gomora J.C., Roger S. (2021). Pharmacological and Nutritional Targeting of Voltage-Gated Sodium Channels in the Treatment of Cancers. iScience.

[176] Bonnin E.A., Golmohammadi A., Rehm R., Tetzlaff C., Rizzoli S.O. (2023). High-Resolution Analysis of Bound Ca^2+^ in Neurons and Synapses. Life Sci. Alliance.

[177] Gleichmann M., Mattson M.P. (2011). Neuronal Calcium Homeostasis and Dysregulation. Antioxid. Redox Signal..

[178] Jang Y.P., Kim S.R., Kim Y.C. (2001). Neuroprotective Dibenzylbutyrolactone Lignans of *Torreya nucifera*. Planta Med..

[179] Kim S.H., Jang Y.P., Sung S.H., Kim C.J., Kim J.W., Kim Y.C. (2003). Hepatoprotective Dibenzylbutyrolactone Lignans of *Torreya nucifera* against CCI4-Induced Toxicity in Primary Cultured Rat Hepatocytes. Biol. Pharm. Bull..

[180] Kim K.Y., Park K.I., Lee S.G., Baek S.Y., Lee E.H., Kim S.C., Kim S.H., Park S.G., Yu S.N., Oh T.W. (2018). Deoxypodophyllotoxin in *Anthriscus sylvestris* Alleviates Fat Accumulation in the Liver via AMP-Activated Protein Kinase, Impeding SREBP-1c Signal. Chem. Biol. Interact..

[181] Gordaliza M., Faircloth G.T., Castro M.A., Miguel Del Corral J.M., López-Vázquez M.L., San Feliciano A. (1996). Immunosuppressive Cyclolignans. J. Med. Chem..

[182] Park H.J., Hong S. (2014). *Anthriscus sylvestris*-Derived Extract Induces Th1 and Th17 Cell Differentiation via the Upregulation of IL12 and IL23 Production. Anim. Cells Syst..

[183] Inamori Y., Kato Y., Kubo M., Baba K., Ishida T., Nomoto K., Kozawa M. (1985). The Biological Actions of Deoxypodophyllotoxin (Anthricin). I. Physiological Activities and Conformational Analysis of Deoxypodophyllotoxin. Chem. Pharm. Bull..

[184] Russell G.B., Singh P., Fenemore P.G. (1976). Insect-Control Chemicals from Plants. III. Toxic Lignans from Lihocedrus hidwillii. Aust. J. Biol. Sci..

[185] Inamori Y., Kato Y., Kubo M., Baba K., Matsuyama Y., Sakai M., Kozawa M. (1983). Mechanisms of Insecticidal Action of Deoxypodophyllotoxin (Anthricin). I.1) Distribution of Deoxypodophyllotoxin in Tissues of the 5th Instar Larvae of Silkworm, *Bombyx mori* LINNE.Pdf. Chem. Pharm. Bull..

[186] Inamori Y., Kato Y., Kubo M., Waku Y., Hayashiya K., Sakai M., Baba K., Kozawa M. (1984). Mechanisms of Insecticidal Action of Deoxypodophyllotoxin (Anthricin). II. Histopathological Studies on Tissues of Silkworm Larvae Intoxicated by Deoxypodophyllotoxin. Chem. Pharm. Bull..

[187] Moss G.P. (2000). Nomenclature of Lignans and Neolignans (IUPAC Recommendations 2000). Pure Appl. Chem..

[188] Terada T., Fujimoto K., Nomura M., Yamashita J., Kobunai T., Takeda S., Wierzba K., Yamada Y., Yamaguchi H. (1992). Antitumor Agents. I. DNA Topoisomerase II Inhibitory Activity and the Structural Relationship of Podophyllotoxin Derivatives as Antitumor Agents. Chem. Pharm. Bull..

[189] Castro A., Del Corral J.M.M., Gordaliza M., Grande C., Gómez-Zurita A., García-Grávalos D., San Feliciano A. (2003). Synthesis and Cytotoxicity of Podophyllotoxin Analogues Modified in the A Ring. Eur. J. Med. Chem..

[190] Gordaliza M., Castro M.A., Miguel del Corral J.M., Lopez-Vazquez M.L., San Feliciano A., Faircloth G.T. (1997). In vivo Immunosuppressive Activity of Some Cyclolignans. Bioorg. Med. Chem. Lett..

[191] Thurston L.S., Imakura Y., Haruna M., Li D., Liu Z., Liu S., Cheng Y., Lee K. (1989). Antitumor Agents. 100. Inhibition of Human DNA Topoisomerase II by Cytotoxic Ether and Ester Derivatives of Podophyllotoxin and Alpha-Peltatin. Am. Chem. Soc..

[192] Van Uden W., Bos J.A., Boeke G.M., Woerdenbag H.J., Pras N. (1997). The Large-Scale Isolation of Deoxypodophyllotoxin from Rhizomes of *Anthriscus sylvestris* Followed by Its Bioconversion into 5- Methoxypodophyllotoxin β-D-Glucoside by Cell Cultures of *Linum flavum*. J. Nat. Prod..

[193] Hu H., Wang Z., Liu S., Cheng Y., Lee K. (1992). Antitumor Agents. 123. Synthesis and Human DNA Topoisomerase II Inhibitory Activity of 2′-Chloro Derivatives of Etoposide and 4&-(Arylamino)-4′-*O*-Demethylpodophyllotoxins. J. Med. Chem..

[194] Brewer C.F., Loike J.D., Horwitz S.B. (1979). Conformational Analysis of Podophyllotoxin and Its Congerers. Structure-Activity Relationships in Microtubule Assembly. J. Med. Chem..

[195] Kelly M.G., Hartwell J.L. (1954). The Biological Effects and the Chemical Composition of Podophyllin. A Review. J. Natl. Cancer Inst..

[196] Rithner C.D., Bushweller C.H. (1983). Dynamic Nuclear Magnetic Resonance and Empirical Force Field Studies of Podophyllotoxin. J. Org. Chem..

[197] Alizadeh B.H., Emami S., Dehghan G., Foroumadi A., Shafiee A. (2017). Synthesis of Cytotoxic Isodeoxypodophyllotoxin Analogs. J. Heterocycl. Chem..

[198] Zhu X., Fu J., Tang Y., Gao Y., Zhang S., Guo Q. (2016). Design and Synthesis of Novel 4′-Demethyl-4-Deoxypodophyllotoxin Derivatives as Potential Anticancer Agents. Bioorg. Med. Chem. Lett..

